# MG53 Coordinates Macrophage Polarization and Neuroimmune Coupling to Promote Corneal Nerve Regeneration via the MPEG1–MVP–STAT6 Axis

**DOI:** 10.1002/advs.202523002

**Published:** 2026-04-10

**Authors:** Peng Chen, Zhentao Zhang, Lilian Sakai, Yanping Xu, Luxi Zhang, Han Ye, William Sollenberger, Takahide Ikeda, Zhiyu Yan, Keerthika Sathish, Usman Alizai, Haitao Wen, Timothy M. Pawlik, Heather L. Chandler, Hua Zhu

**Affiliations:** ^1^ Department of Surgery The Ohio State University Wexner Medical Center Columbus Ohio USA; ^2^ College of Optometry The Ohio State University Columbus Ohio USA; ^3^ Department of Microbial Infection and Immunity The Ohio State University Columbus Ohio USA

**Keywords:** corneal nerve regeneration, corneal wound healing, macrophage, MG53, MPEG1, MVP

## Abstract

Corneal sensory nerve plays a critical role in pain sensation and corneal wound healing. However, the molecular mechanisms that regulate corneal nerve repair, especially couple immune regulation to corneal nerve repair remain poorly understood. Here, Mitsugumin 53 (MG53), a E3 ubiquitin ligase, is identified as a pivotal regulator of macrophage‐mediated corneal nerve regeneration. MG53 is present in tear film, aqueous humor, and corneal epithelial cells, suggesting its role in corneal homeostasis. In an alkali burn injury model, genetic ablation of MG53 impairs nerve regrowth, whereas genetic overexpression or delivery of MG53 modified RNA markedly enhanced corneal nerve regeneration. Mechanistically, MG53 interacts with major vault protein (MVP) and promotes its K63‐linked ubiquitination at lysine 747, facilitating STAT6 nuclear translocation and transcriptionally activating M2 (reparative) macrophage genes. MG53 overexpression biases macrophage polarization toward a reparative phenotype characterized by elevated Arg1, Fizz1, Ym1/2, and IRF4 expression, thereby enhancing clearance of degenerating nerve fragments and promoting nerve regeneration. Using a genome‐wide Membrane Proteome Array (MPA) screen, macrophage‐expressed gene 1 (MPEG1) is identified as a putative receptor mediating MG53 internalization in macrophages. These findings establish a mechanistic framework in which circulating MG53 engages the MPEG1–MVP–STAT6 axis to coordinate macrophage polarization and neuroimmune repair.

## Graphical abstract

Corneal nerve regeneration is critical to corneal wound healing processes. The current study reveals a novel role of MG53 in promoting corneal nerve regeneration after alkali induced injury. Mechanistically, MG53 enters macrophages via its receptor, MPEG1, promotes MVP K63 ubiquitination, and triggers STAT6 induced repair‐related genes expression, which is essential for post‐injury corneal nerve regeneration.

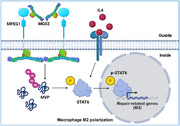

## 1 Introduction

The cornea is the window of our eye and is among the most densely innervated tissue of the body. In physiological conditions, corneal sensory nerves sense pain and regulate tearing and blink reflexes to prevent corneal injuries [1] In response to traumatic injury, infection, and ocular surgery, corneal nerves regulate ocular surface cells, such as limbal stem cells (LSCs) [2, 3, 4, 5], corneal epithelial cells (CEC) [6, 7], and keratocytes [6, 8], to promote corneal wound healing. If the sensory nerve network is lost in pathological conditions, the cornea can undergo repetitive non‐healing ulceration, develop corneal opacification, limbal stem cell deficiency (LSCD), and even permanent blindness [9, 10, 11].

Recent findings further support the notion that multiple cellular and molecular factors act in concert to determine the outcome of corneal wound healing. For example, topical application of recombinant human nerve growth factor (rhNGF) not only promotes corneal nerve regeneration, but also accelerates epithelial healing and modulates angiogenesis following alkali injury [12]. Moreover, accumulating evidence indicates that NGF is a key regulator of ocular surface homeostasis and repair. Beyond its classical neurotrophic functions, NGF influences epithelial proliferation, immune responses, tear secretion, and angiogenesis, and has shown therapeutic potential in a broad spectrum of ocular surface disorders, including neurotrophic keratitis, herpetic keratitis, dry eye disease, and post‐surgical corneal healing [13]. Unfortunately, this drug is extremely expensive, and due to the short half‐life of the recombinant protein, daily application is required. Further, not all patients respond to rhNGF treatment [14]. Thus, more studies are required to uncover the cellular and molecular pathways associated with corneal nerve repair and regeneration, particularly to dissect the crosstalk between different corneal cells to maintain corneal nerve integrity and facilitate regeneration. Strategies that integrate these aspects may provide new preventive and therapeutic avenues to restore both structural integrity and visual function after corneal injury.

Mitsugumin 53 (MG53), is a tripartite motif‐containing protein (TRIM72) that functions as an essential component of the cell membrane repair machinery, serving as a molecular bandage to facilitate rapid resealing of injured membranes across multiple tissues [15, 16, 17]. As a muscle‐enriched E3 ubiquitin ligase, MG53 rapidly oligomerizes upon oxidative stress and recruits intracellular vesicles to sites of membrane disruption, thereby preserving cellular integrity under various forms of injury [18] Beyond its canonical role in membrane repair, accumulating evidence suggests that MG53 exerts broad cytoprotective effects through its anti‐inflammatory and anti‐fibrotic actions in cardiac [19, 20, 21, 22, 23, 24], pulmonary [25, 26, 27], renal [28, 29], and skeletal muscle injury [30, 31, 32], models. Recombinant human MG53 (rhMG53) administration has been shown to mitigate ischemic damage [33], suppress inflammatory responses [34], and promote tissue regeneration [35] in several organ systems.

Our previous research has demonstrated that MG53 facilitates rapid repair of corneal wounds and reduces fibrotic remodeling and vascularization, supporting its role in maintaining corneal transparency [36, 37]. Most recently, we also observed that MG53 can maintain corneal nerve density after alkali‐induced corneal injury, suggesting a previously unrecognized role in modulating neuro‐regeneration within the corneal microenvironment [38] However, the underlying molecular mechanisms governing MG53‐mediated corneal nerve protection and regeneration remain unclear. In the present study, we aimed to elucidate the functional role of MG53 in this pathological process and to define how it contributes to coordinated epithelial, stromal, and neural regeneration following corneal injury.

## 2 Results

### 2.1 MG53 Promotes Corneal Nerve Regeneration Following Alkali Injury in Vivo

**FIGURE 1:**
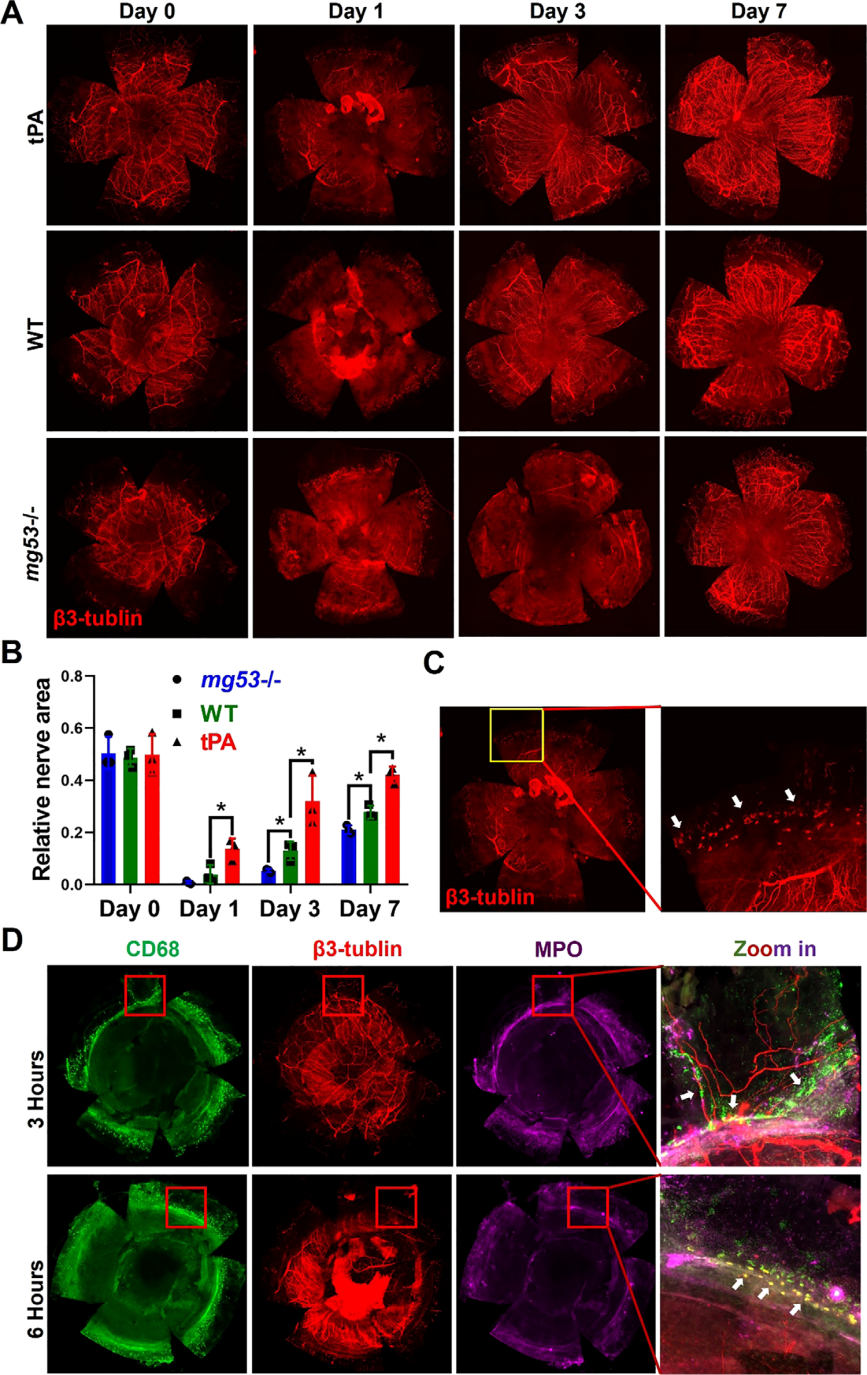
MG53 promotes corneal nerve regeneration after alkali injury. (A) Whole‐mount immunostaining of β3‐tubulin (red) showing corneal nerve regeneration at indicated time points (Day 0, 1, 3, and 7) after alkali injury in tPA‐MG53, WT, and *mg53‐/‐* mice. Nerve regeneration was accelerated in tPA‐MG53 mice and impaired in *mg53‐/‐* mice. (B) Quantification of relative nerve area during corneal healing across genotypes. (C) Representative β3‐tubulin staining image showing regenerating corneal nerves (white arrows) extending toward the injury center. (D) Immunofluorescence staining of corneal whole mounts showing infiltration of CD68^+^ macrophages (green) and MPO^+^ neutrophils (purple) at 3–6 h post‐injury, co‐localizing with regenerating β3‐tubulin^+^ nerve fibers (red). Data are presented as mean ± SD (*n* = 3 per group). ^*^ *p* < 0.05.

First, we established an alkali‐induced corneal injury model using MG53 global knockout (*mg53‐/‐*) mice, transgenic mice with elevated MG53 in the systemic blood circulation (tPA‐MG53) [39], and wild type (WT) littermate mice. Corneal flat‐mount staining was performed on 0, 1, 3, and 7 days post‐injury to evaluate nerve density and regeneration. As shown in Figure 1A, corneal nerve structure and morphology remained largely intact immediately following alkali injury. However, by day 1, corneal nerve density was significantly reduced (*p* < 0.001, WT day 1 compared with day 0). Nerve regrowth began thereafter, and tPA‐MG53 mice showed significantly enhanced nerve regeneration and corneal nerve density compared to WT littermates (day 1, *p* = 0.034; day 3, *p* = 0.036; day 7, *p* = 0.003) (Figure 1B). In contrast, *mg53‐/‐* mice exhibited significantly reduced corneal nerve density (day 3, *p* = 0.022; day 7, *p* = 0.017). By day 7, corneal nerve density in tPA‐MG53 mice had nearly returned to pre‐injury levels, whereas *mg53‐/‐* mice still displayed significantly lower nerve density than WT controls (*p* = 0.017) (Figure 1B). Interestingly, we observed that at Day 1 post‐injury, the significant reduction in corneal nerve density was accompanied by the appearance of numerous β3‐tubulin‐positive puncta at the limbal region (arrows in Figure 1C).

To determine the timing and identity of these puncta, we narrowed the observation window and performed immunostaining at 3‐ and 6‐h post‐injury. As shown in Figure 1D, while no puncta were present, CD68‐positive cells were already observed accumulating around corneal nerve fibers in the limbus as early as 3 h after injury (Figure S1). By 6 h, β3‐tubulin‐positive puncta became clearly detectable and exhibited distinct co‐localization with CD68‐positive cells but not with MPO positive cells (Figure 1D; Figure S1). These findings suggest that CD68‐positive cells might respond to the local injury microenvironment by engaging in the clearance of degenerating corneal nerves (β3‐tubulin‐positive cells puncta). This speculative interpretation could help explain why corneal nerves appear relatively intact during the early phase after injury but are nearly completely absent by 24 h.

To further identify the cell type exhibiting co‐localization of CD68 and β3‐tubulin, we performed additional immunofluorescence staining using a panel of lineage‐specific markers. As shown in Figures S2 and S3, these cells did not co‐localize with CD80 or CD192, markers of pro‐inflammatory macrophages. However, they exhibited robust co‐localization with CD206, CD163, and CD11b, markers often associated with a “repair‐associated” or anti‐inflammatory macrophage phenotype. With higher magnification imaging, β3‐tubulin is clearly observed within the CD68^+^ and CD206^+^ cells (Figure S4). The β3‐tubulin signal appears as punctate aggregates localized inside the cytoplasm of the CD68^+^ cell, rather than remaining on the cell surface or extracellularly. This spatial distribution is consistent with internalization of neuronal fragments into the intracellular compartment of macrophages from other published studies [40], potentially reflecting a role in the clearance of degenerating corneal nerve elements.

### 2.2 M2‐Type Macrophages Mediate Early Corneal Nerve Clearance Following Injury

As shown in Figure S5, distinct differences presented in immune cell infiltration and nerve regeneration among the genotypes. At day 2 after injury, CD206^+^ macrophages were significantly reduced in *mg53‐/‐* corneas compared to WT corneas (*p* = 0.044), while tPA‐MG53 corneas had significantly increased number of CD206^+^ cells within the injured area (*p* = 0.013). β3‐tubulin staining revealed significant nerve loss in *mg53‐/‐* corneas (*p* = 0.016), with only sparse nerve fibers remaining in the central cornea. In contrast, WT corneas showed partial nerve regeneration, while tPA‐MG53 mice had more robust and organized nerve recovery across the wound area. These observations showed a positive correlation between MG53 expression, M2 (reparative) macrophage, and post‐injury corneal nerve regeneration.

**FIGURE 2:**
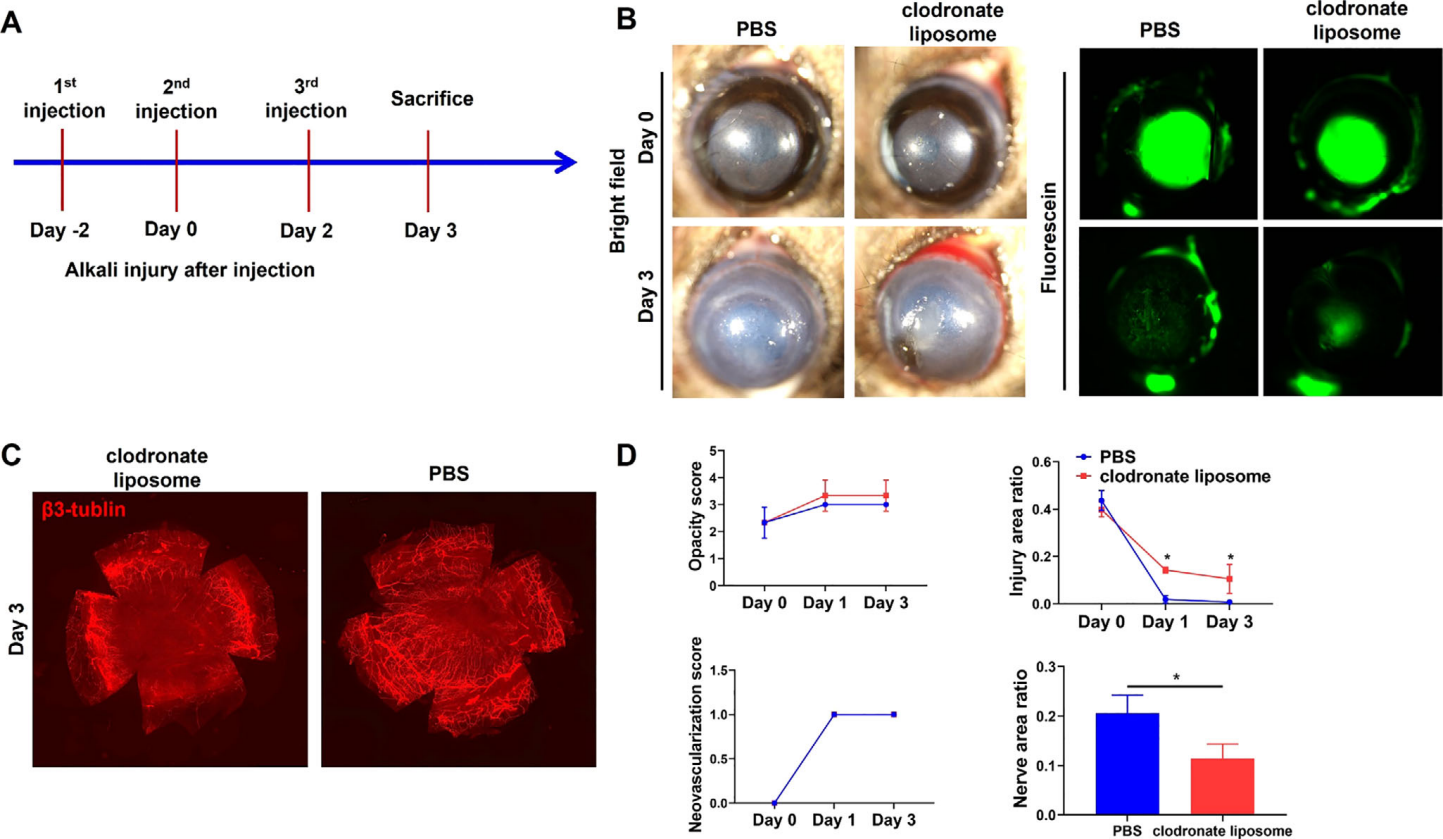
Depletion of macrophages impairs corneal nerve regeneration after alkali injury.(A) Experimental timeline. Mice received intravenous injections of clodronate or PBS liposomes on Day −2, Day 0, and Day 2. Corneal alkali injury was induced after the second injection, and eyes were collected on Day 3 for analysis. (B) Representative bright‐field and fluorescein staining images of injured corneas treated with PBS or clodronate liposomes at Day 0 and Day 3. Corneal opacity and epithelial wound healing were evaluated over time. (C) Whole‐mount immunostaining of corneal β3‐tubulin (red) showing corneal nerve regeneration at Day 3 after alkali injury in PBS‐ or clodronate‐treated mice. (D) Quantification of corneal opacity score, injury area ratio, and neovascularization score at indicated time points. The bar graph (bottom right) shows quantification of corneal nerve area ratio at Day 3. Data are presented as mean ± SD (*n* = 3 per group). ^*^ *p* < 0.05.

To further investigate the potential role of macrophages in corneal nerve regeneration, we used clodronate liposomes to deplete macrophages in mice before and after corneal injury (Figure 2A). An alkali injury was then created; corneal fluorescein staining was performed daily to evaluating epithelial healing and corneal flat‐mount staining was performed on day 3 post‐injury. The results show that macrophage depletion led to delayed corneal epithelial healing and a significant reduction in corneal nerve density (Figure 2B–D), supporting the role of macrophages in promoting post‐injury corneal nerve regeneration.

### 2.3 MG53 Promotes Nuclear Translocation of MVP and BMDM Polarization

**FIGURE 3:**
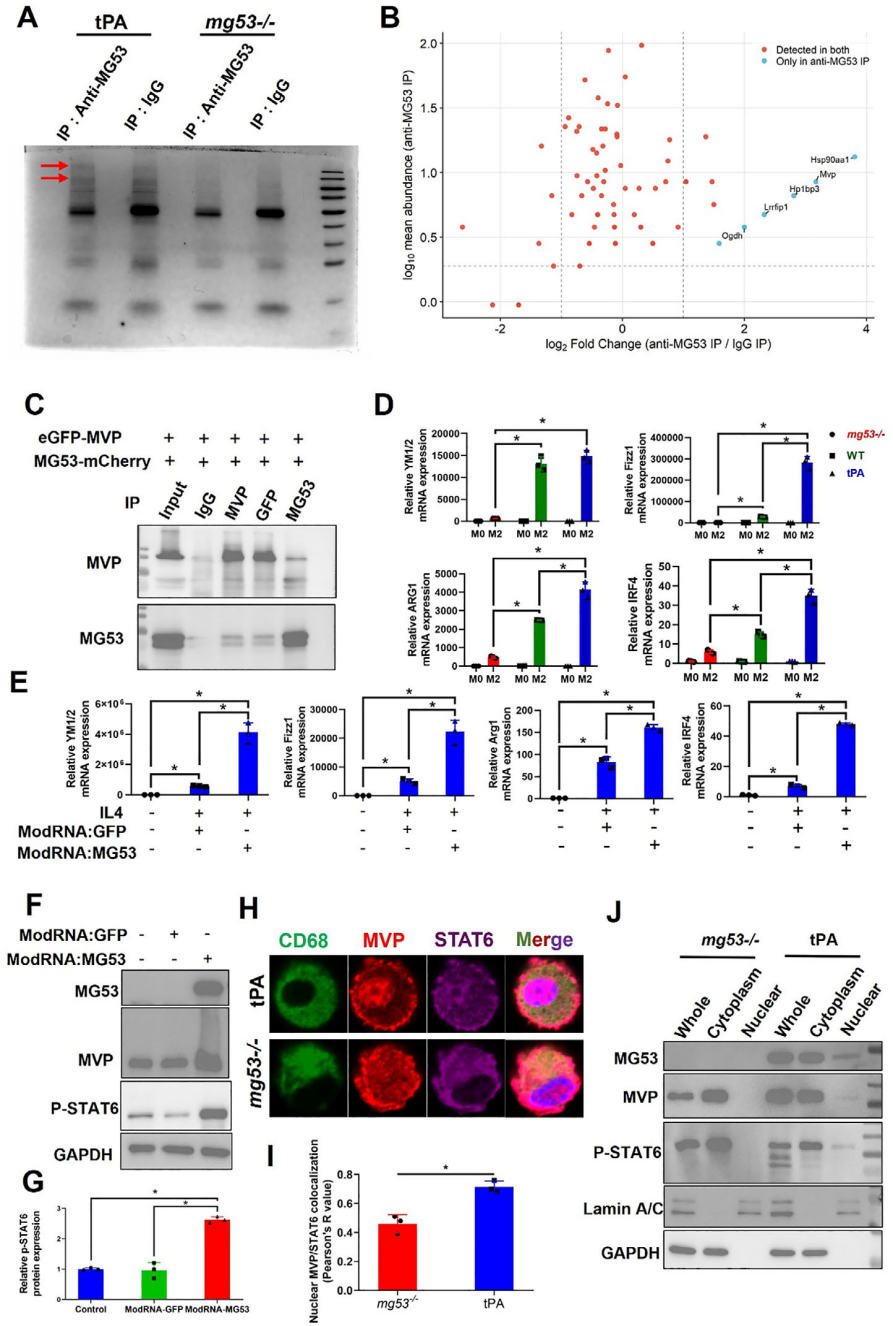
MG53 interacts with MVP to regulate STAT6 activation during macrophage M2 polarization. (A) Coomassie staining of proteins co‐immunoprecipitated with MG53 from tPA‐MG53 and *mg53^−^/^−^* mouse macrophage (M2) lysates. Red arrows indicate protein bands enriched in the tPA‐MG53 group that were absent in *mg53^−^/^−^* controls. (B) Volcano plot of LC–MS/MS proteomic analysis showing proteins enriched in MG53 immunoprecipitates. MVP was identified as one of the top MG53‐interacting candidates. (C) Co‐immunoprecipitation of MG53‐mCherry and eGFP‐MVP in HEK293T cells confirming the MG53–MVP interaction. (D) Quantitative PCR analysis showing mRNA expression of M2‐related genes (Ym1/2, Fizz1, Arg1, and IRF4) in BMDMs from WT, mg53^−^/^−^, and tPA‐MG53 mice after IL‐4 stimulation. Data are presented as mean ± SD (*n* = 3 per group). (E) Expression of M2 marker genes in BMDMs transfected overnight with ModRNA‐GFP or ModRNA‐MG53, followed by IL‐4 stimulation for 24 h. Data are presented as mean ± SD (*n* = 3 per group). (F) Western blot analysis of MG53, MVP, STAT6, and phospho‐STAT6 in BMDMs transfected overnight with ModRNA‐GFP or ModRNA‐MG53, then treated with IL‐4 for 24 h. (G) Quantification of phospho‐STAT6 levels are presented as mean ± SD from three independent experiments shown in (F). (H) Immunofluorescence staining of M2‐polarized CD68^+^ macrophages (green) showing co‐localization of MVP (red) and STAT6 (magenta). (I) Quantification of nuclear MVP/STAT6 colocalization in macrophages. Regions of interest (ROIs) were defined based on DAPI‐positive nuclear areas, and colocalization between MVP and STAT6 signals within the nuclear compartment was analyzed using Pearson's correlation coefficient (R value). (*n* = 3 per group). Data are presented as mean ± SD. (I) Subcellular fractionation showing cytoplasmic and nuclear distribution of MVP and p‐STAT6 in *mg53^−^/^−^* and tPA‐MG53 macrophages (M2). Lamin A/C and GAPDH served as nuclear and cytoplasmic markers, respectively.

To further investigate how MG53 regulates macrophage polarization, we performed co‐immunoprecipitation (Co‐IP) using an anti‐MG53 antibody in IL‐4 induced bone marrow derived macrophages (M2 BMDMs) derived from tPA‐MG53 and *mg53‐/‐* mice. As shown by Coomassie blue staining, compared to both the *mg53‐/‐* and IgG pull‐down groups, the MG53 antibody pull‐down sample from tPA‐MG53 mice displayed distinct protein bands in the 70–180 kDa range (Figure 3A). These specific bands were excised for subsequent mass spectrometry analysis. Mass spectrometry analysis revealed the presence of unique potential interacting proteins in the MG53 antibody pull‐down group (Figure 3B). Subsequent co‐immunoprecipitation and immunoblotting were performed, which confirmed a strong interaction between MG53 and major vault protein (MVP) (Figure 3C).

Interestingly, previous studies have reported that MVP is involved in macrophage polarization [41, 42]. MVP has been shown to promote the phosphorylation and nuclear translocation of STAT6, thereby enhancing the expression of IRF4, which in turn upregulates downstream M2‐associated genes, such as Fizz1, Ym1/2, and Arg1 [41, 42].

Next, we sought to determine whether MG53 influences macrophage polarization. We isolated bone marrow cells from WT, *mg53‐/‐*, and tPA‐MG53 mice and differentiated the cells into macrophages (BMDMs). These BMDMs were then stimulated with IL‐4 to induce M2 polarization. Twenty‐four hours after IL‐4 treatment, expression of M2 macrophage markers was significantly upregulated compared to unstimulated (M0) controls. However, the magnitude of this upregulation varied significantly among the different genotypes. In BMDMs from tPA‐MG53 mice, expression of Arg1 (*p* = 0.002) and Fizz1 (*p* < 0.001) was significantly higher than in WT BMDMs. In contrast, BMDMs from *mg53‐/‐* mice showed significantly lower upregulation of Arg1 (*p* < 0.001), Fizz1 (*p* < 0.001) and YM1/2 (*p* < 0.001) compared to WT controls (Figure 3D). We further examined the expression of IRF4, a transcription factor associated with M2 polarization. Consistent with previous results, IRF4 expression was significantly higher in BMDMs from tPA‐MG53 mice compared to WT controls (*p* < 0.001), whereas *mg53‐/‐* BMDMs showed reduced IRF4 upregulation relative to WT mice (*p* < 0.001) (Figure 3D).

To validate our finding that MG53 modulates macrophage polarization and study the potential role of MG53 in MVP‐mediated polarization, we overexpressed MG53 with MG53 modified RNA (MG53 modRNA) in BMDMs derived from *mg53‐/‐* mice. The results show that MG53 overexpression significantly increased the IL‐4–induced upregulation of Arg1 (*p* < 0.001), Fizz1 (*p* = 0.0017), Ym1/2 (*p* < 0.001), and IRF4 (*p* < 0.001) compared to the control group (Figure 3E). Further, we demonstrate that MG53 overexpression significantly increased the phosphorylation level of STAT6 (Figure 3F,G). We also observed that MG53 overexpression led to the appearance of smear bands for MVP, suggesting potential post‐translational modifications (Figure 3F). Immunofluorescence staining confirmed that in M2‐polarized BMDMs derived from tPA‐MG53 mice, MVP was predominantly localized in the nucleus. In contrast, in BMDMs from *mg53‐/‐* mice, MVP was evenly distributed throughout the cytoplasm. Similarly, STAT6 showed strong nuclear accumulation in M2 BMDMs from tPA‐MG53 mice, whereas in *mg53‐/‐* BMDMs, STAT6 remained largely cytoplasmic with minimal nuclear localization (Figure 3H,I). We further performed nuclear and cytoplasmic fractionation of BMDMs from mice with different genotypes. The results show that in tPA‐MG53 BMDMs, both MVP and phosphorylated STAT6 (p‐STAT6) were able to translocate into the nucleus. In contrast, in *mg53‐/‐* BMDMs, nuclear localization of MVP and p‐STAT6 was barely detectable (Figure 3J). Taken together, these results suggest that MG53 interacts with MVP and promotes M2 (reparative) macrophage polarization.

### 2.4 MG53 Promotes MVP K63 Ubiquitination on the K747 Residue

**FIGURE 4:**
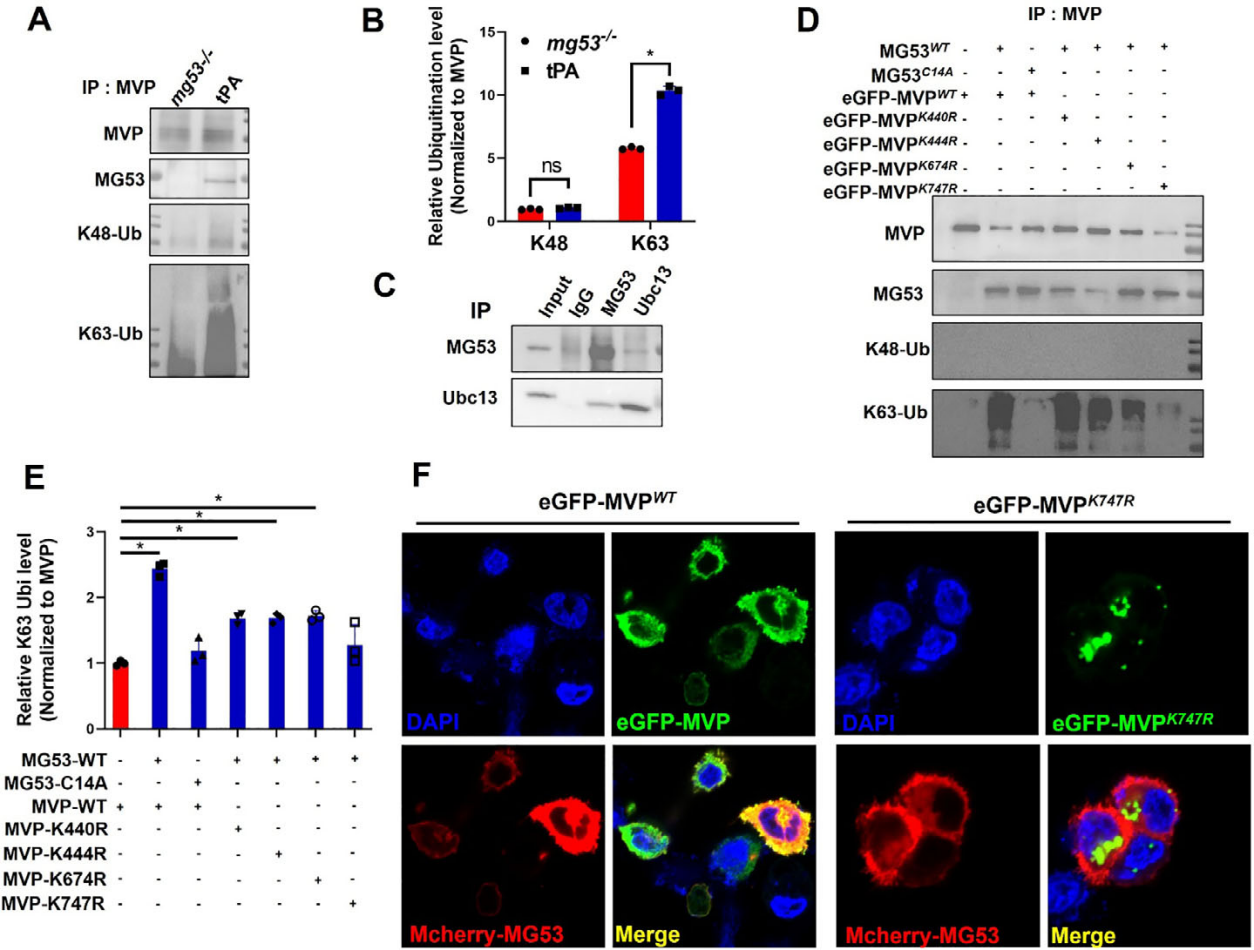
MG53 promotes MVP ubiquitination through its E3 ligase activity. (A) Co‐immunoprecipitation (IP) analysis of MVP ubiquitination in M2‐polarized bone marrow–derived macrophages (BMDMs) isolated from *mg53^−^/^−^* and tPA‐MG53 mice. MVP was immunoprecipitated and immunoblotted for MG53, K48‐linked ubiquitin, and K63‐linked ubiquitin. (B) Quantification of K48‐ and K63‐linked ubiquitination levels shown in (A). Ubiquitination signals were quantified by densitometry and normalized to total immunoprecipitated MVP. Data are presented as mean ± SD from three independent experiments. ^*^ *p* < 0.05; ns, not significant. (C) Reciprocal co‐IP showing the interaction between MG53 and the E2 enzyme Ubc13 in M2 BMDMs from tPA‐MG53 mice. (D) Ubiquitination assays in HEK293T cells co‐expressing MG53‐WT or the MG53‐C14A with MVP‐WT or lysine mutants (K440R, K444R, K674R, K747R). MVP was immunoprecipitated and probed with antibodies against MVP, MG53, K48‐ and K63‐linked ubiquitin. (E) Quantification of K63‐linked ubiquitination levels shown in (D), normalized to total MVP. Data represent mean ± SD from three independent experiments. ^*^p < 0.05. (F) Confocal microscopy showing the subcellular localization of mCherry‐MG53 (red) with eGFP‐MVP or eGFP‐MVP‐K747R (green) in HEK293T cells. Nuclei were stained with DAPI (blue). MG53 co‐localizes with wild‐type MVP in the cytoplasm, while the K747R mutation reduces this interaction.

Given that MG53 functions as an E3 ubiquitin ligase and the observation of MVP smear bands after MG53 overexpression (Figure 3F), we hypothesized that MG53 might trigger ubiquitination of MVP. We performed co‐immunoprecipitation using an anti‐MVP antibody in BMDMs from *mg53‐/‐* and tPA‐MG53 mice. The results show that MVP was able to pull down MG53, and that MVP underwent K63‐linked ubiquitination, rather than K48‐linked ubiquitination in tPA‐MG53 BMDMs (Figure 4A,B). Unlike K48‐linked ubiquitination mediated protein degradation, K63‐linked ubiquitination usually alters subcellular localization of its substrates [43, 44]. In addition, there are reports demonstrating that MG53 can interact with the E2 ubiquitin‐conjugating enzyme Ubc13, which is specifically involved in catalyzing K63‐linked ubiquitination [45, 46]. Indeed, we confirmed there are interaction between MG53 and Ubc13 in BMDMs from tPA‐MG53 mice (Figure 4C).

To further identify the specific lysine residues in MVP that undergo K63‐linked ubiquitination by MG53, we used PhosphoSitePlus to analyze potential ubiquitination sites reported in high‐throughput studies [47]. Based on the frequency of reported modifications, we selected four candidate lysine residues—K440, K444, K674, and K747—and generated corresponding point mutants (K440R, K444R, K674R, and K747R) by site‐directed mutagenesis. These plasmids were co‐transfected into HEK293 cells along with either WT MG53 or the E3 ligase‐deficient MG53 mutant (C14A) plasmids. The results show that the C14A mutant, which disrupts MG53's E3 ligase activity, failed to induce K63‐linked ubiquitination of MVP (Figure 4D,E). Among the MVP mutants, K440R, K444R, and K674R variants still underwent K63‐linked ubiquitination in the presence of WT MG53. However, the K747R mutant completely lost its ability to be ubiquitinated, indicating that K747 is a critical site for MG53‐mediated K63‐linked ubiquitination of MVP (Figure 4D,E). Since K63‐linked ubiquitination usually controls the subcellular localization of the substrates, we next performed live cell imaging analysis. Interestingly, the K747R mutation markedly altered the subcellular distribution of MVP. The mutant (K747R) no longer co‐localized with MG53 and showed significantly reduced nuclear localization compared to WT MVP (Figure 4F). These results demonstrated that MG53 ubiquitinated MVP at K747 residue, facilitating translocation to the nucleus for downstream STAT6 signal cascade.

To further determine whether STAT6 activation is required for MG53‐mediated neuroprotective effects in vivo, we employed the STAT6 inhibitor PM43I in a corneal injury model [48]. Mice were treated with PM43I before and following injury, and corneal epithelial healing, neovascularization, and nerve regeneration were evaluated. As shown in Figure S6, Pharmacological inhibition of STAT6 impaired corneal repair in a stage‐dependent manner. At Day 1 post‐injury, PM43I‐treated mice exhibited significantly delayed epithelial wound closure compared with vehicle‐treated controls. Although no significant differences were observed from Day 3 onward, corneas in the PM43I‐treated group developed recurrent epithelial defects by Day 10, whereas control corneas remained fully re‐epithelialized. In addition, STAT6 inhibition enhanced corneal neovascularization and markedly reduced corneal nerve fiber density at later stages of healing.

We then evaluated corneal nerve integrity in WT and tPA‐MG53 mice following PM43I treatment. As shown in Figure S7A–D, CD68^+^ macrophages were still present at the corneal limbus at 3 and 6 h post‐injury in both WT and tPA‐MG53 mice after STAT6 inhibition. However, the spatial co‐localization between CD68 and β3‐tubulin was markedly reduced, suggesting impaired macrophage–nerve interaction under STAT6 blockade. Consistently, quantitative analysis at Day 7 (Figure S8A–B) demonstrated that PM43I treatment significantly reduced corneal nerve regeneration in both WT and tPA‐MG53 mice compared with their respective untreated controls. Notably, despite STAT6 inhibition, tPA‐MG53 mice still exhibited significantly greater nerve preservation than WT mice. These findings indicate that while STAT6 signaling contributes substantially to MG53‐mediated nerve repair, MG53 may also promote corneal nerve regeneration through additional STAT6‐independent mechanisms.

### 2.5 Therapeutic Efficacy of MG53 Modified RNA via Both Local and Systemic Delivery

In our previous work, we reported that topical application of rhMG53 protein to the eye could promote corneal wound healing [36, 37, 38]. However, multiple applications per day were required due to the limited half‐life of rhMG53 (∼2 h) and rapid clearance by tears on the ocular surface. Modified RNA (modRNA) offers the advantage of sustained endogenous MG53 expression with fewer administrations. It enables efficient protein production within target cells, avoids genomic integration, and, with proper chemical modifications, minimizes immune activation, making modRNA a promising strategy for corneal therapy [49]. Therefore, we designed and synthesized modRNAs encoding either MG53 or GFP (as control), and directly injected them into the mouse corneal stroma.

**FIGURE 5:**
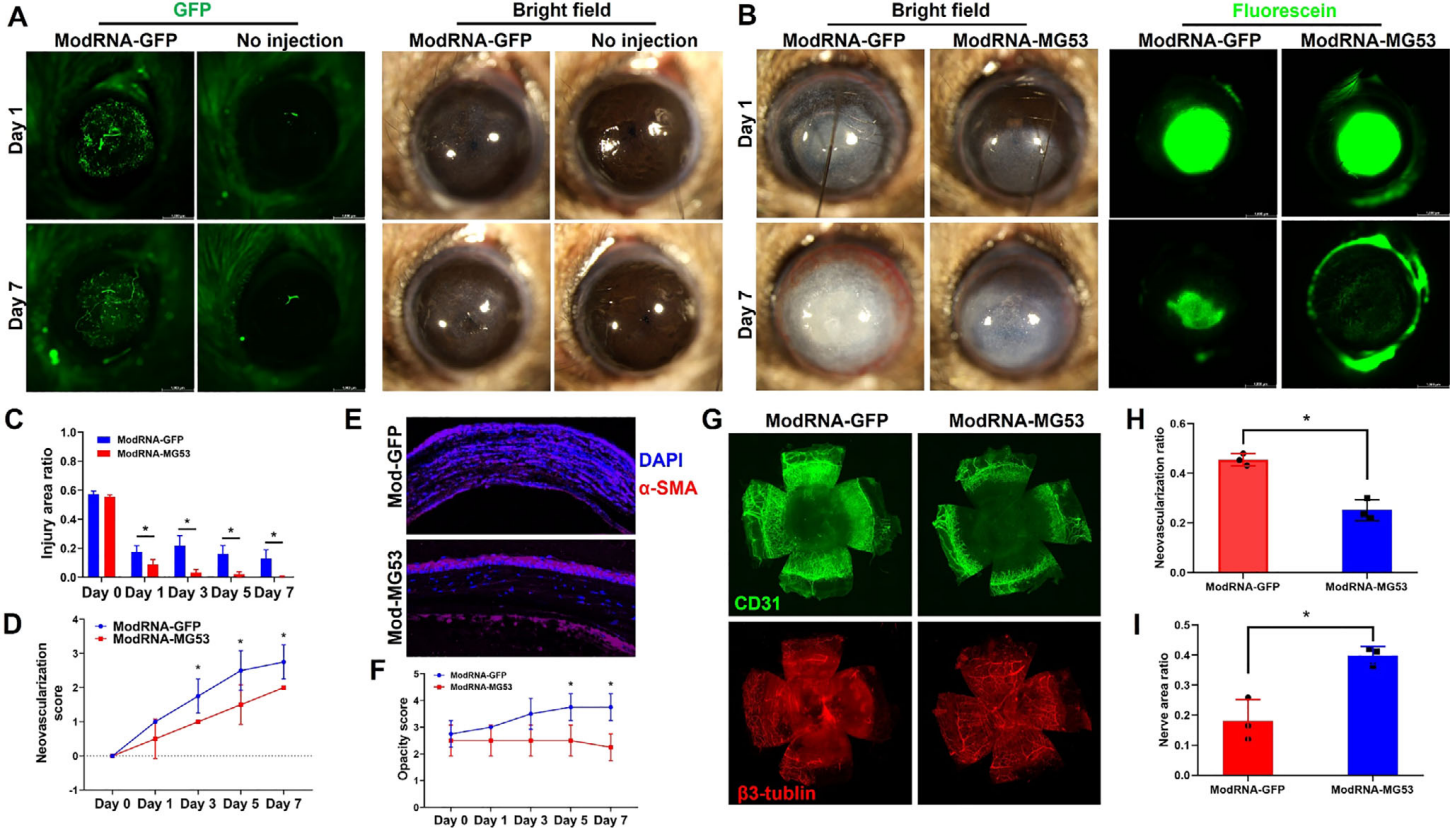
ModRNA‐mediated MG53 delivery promotes corneal wound healing and nerve regeneration after alkali injury. (A) Representative GFP fluorescence and bright‐field images showing successful corneal transfection after local injection of ModRNA‐GFP. GFP expression was detectable in the corneal epithelium at Day 1 and persisted through Day 7. (B) Representative bright‐field and fluorescein staining images of injured corneas in ModRNA‐GFP and ModRNA‐MG53–treated mice at Day 1 and Day 7. (C,D) Quantification of injury area ratio (C) and neovascularization score (D) over time. (E) Quantification of corneal opacity score over time. (F) Immunofluorescence staining of α‐SMA (red) and DAPI (blue) in corneal cross‐sections at Day 7, showing reduced stromal fibrosis in ModRNA‐MG53–treated eyes. (G) Whole‐mount corneal staining for CD31 (green) and β3‐tubulin (red) at Day 7, showing decreased neovascularization and enhanced nerve regeneration in ModRNA‐MG53–treated corneas compared with ModRNA‐GFP controls. Quantification of CD31^+^ neovascular area (H) and β3‐tubulin^+^ nerve area (I) at Day 7. Data are presented as mean ± SD (*n* = 4 per group). ^*^ *p* < 0.05.

We first confirmed the duration of gene expression following modRNA injection into the cornea. As shown in Figure 5A, a single injection of GFP modRNA resulted in stable expression lasting up to 7 days. We next investigated whether delivery of MG53 modRNA could provide protective effects against ocular injury. Mice were injected intrastromally with modRNA encoding either GFP or MG53, followed by induction of an alkali injury. As shown in Figure 5B–I, mice treated with MG53 modRNA accelerated corneal wound healing (Figure 5B,C), reduced fibrosis (Figure 5E,F), reduced neovascularization (Figure 5D,G,H), and improved nerve regeneration (Figure 5G,I).

However, as observed in Figure 5B, the residual buffer from the modRNA injection was not fully absorbed, leading to suboptimal corneal transparency despite lower expression of fibrotic markers in MG53 modRNA treated mice. To address this issue, we adopted an alternative strategy by constructing modRNA encoding HA‐tagged tPA‐MG53 and delivering it via intramuscular injection. We have successfully used tPA modification to facilitate secretion of MG53 into the bloodstream in our transgenic mouse model (tPA‐MG53 mice) [39]. Here, we hoped that intramuscular injection of tPA‐MG53 modRNA could elevate circulating MG53 level and allow it to reach the cornea through the limbal vasculature.

**FIGURE 6:**
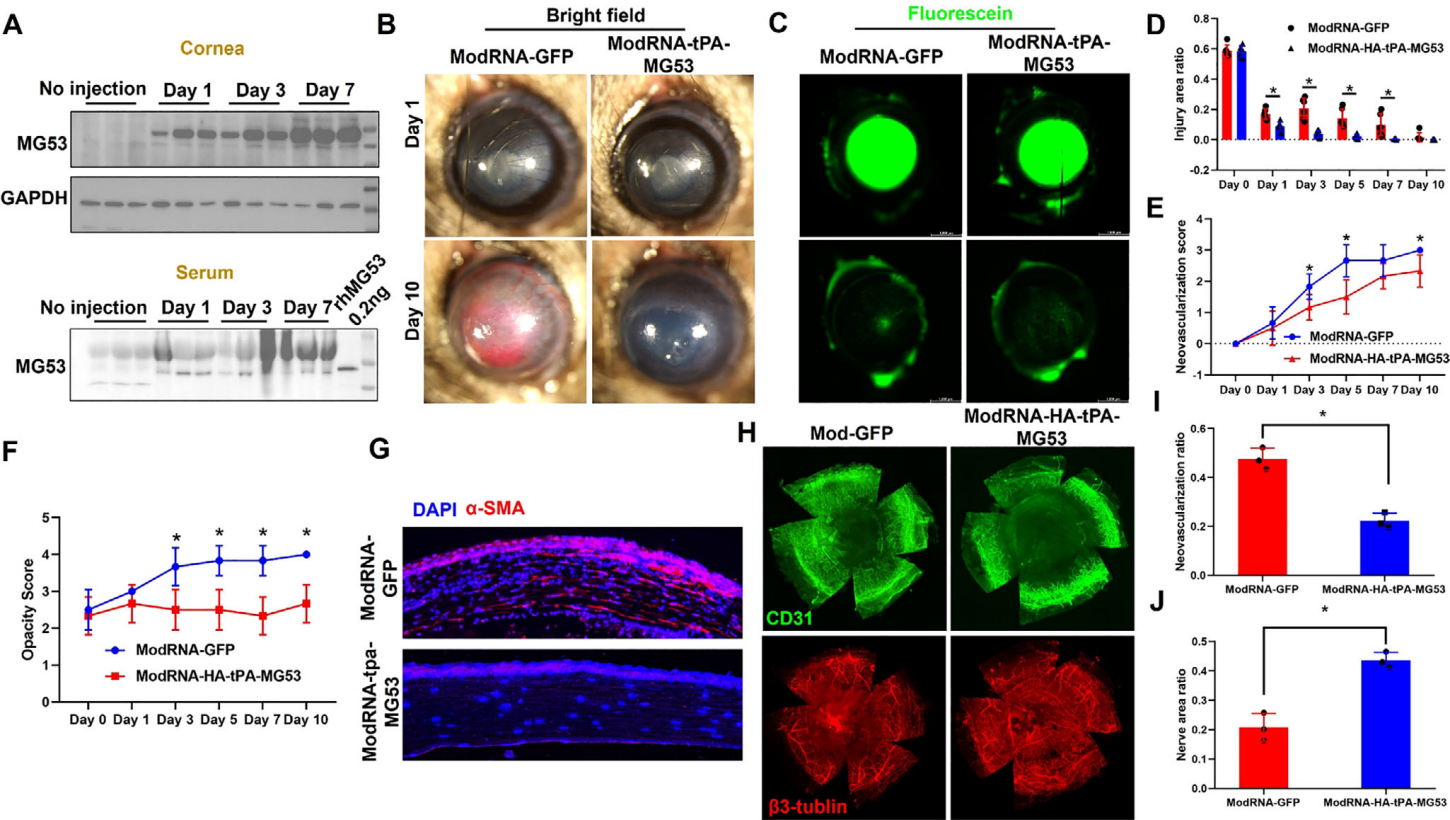
Systemic delivery of ModRNA‐HA‐tPA‐MG53 enhances corneal wound repair and nerve regeneration after alkali injury. (A) Western blot analysis of MG53 protein expression in corneal tissue and serum at the indicated time points after intravenous administration of ModRNA‐HA‐tPA‐MG53 or ModRNA‐GFP. MG53 levels increased in both cornea and serum, confirming successful systemic delivery. (B,C) Representative bright‐field (B) and fluorescein staining (C) images showing improved corneal clarity and epithelial wound closure in ModRNA‐HA‐tPA‐MG53–treated mice compared with ModRNA‐GFP controls. (D–F) Quantification of corneal injury area ratio (D), opacity score (E), and neovascularization score (F) over time. (G) Immunofluorescence staining of α‐SMA (red) and DAPI (blue) in corneal cross‐sections at Day 10, showing reduced stromal fibrosis in ModRNA‐HA‐tPA‐MG53–treated eyes. (H) Whole‐mount corneal staining for CD31 (green) and β3‐tubulin (red) at Day 10, demonstrating reduced neovascularization and enhanced nerve regeneration following ModRNA‐HA‐tPA‐MG53 treatment. (I,J) Quantification of CD31^+^ neovascular area (I) and β3‐tubulin^+^ nerve area (J) at Day 10. Data are presented as mean ± SD (*n* = 6 per group). ^*^ *p* < 0.05.

As shown in Figure 6A and Figure S9, intramuscular injection of tPA‐MG53 modRNA in mice resulted in a marked increase in MG53 levels in both serum and corneal tissue 1, 3, and 7 days post‐injection. Figure 6B shows representative bright‐field images of injured corneas, where tPA‐MG53 modRNA‐treated mice exhibited markedly improved corneal clarity compared to the control group at both day 1 and day 10. Figure 6C–F demonstrate that tPA‐MG53 modRNA treatment significantly reduced the corneal injury area ratio (day 1, *p* = 0.0014; day 3, *p* < 0.001; day 5, *p* < 0.001; day 5, *p* < 0.001; day 7, *p* < 0.001), clinically visible vascular encroachment (day 3, *p* = 0.017; day 5, *p* = 0.0035; day 10, *p* = 0.01), and corneal opacity (day 3, *p* = 0.0035; day 5, *p* < 0.001; day 7, *p* < 0.001; day 10, *p* < 0.001), respectively. Histological analysis (Figure 6G) revealed that α‐SMA expression, a marker of fibrosis, was substantially reduced in corneas from modRNA MG53 treated mice. Flat‐mount immunostaining (Figure 6H) confirmed a reduction in CD31^+^ vascularization (*upper panels*) and a corresponding improvement in β3‐tubulin^+^ nerve fiber density (*lower panels*). Quantification of these parameters (Figure 6I,J) confirms a significant decrease in neovascularization ratio (*p* = 0.0012) and an increase in corneal nerve area ratio (*p* = 0.0018) in the MG53 modRNA treated group, compared to GFP modRNA treated mice.

### 2.6 MPEG1 Serves as a Putative Receptor for MG53 in Macrophages

Our above results demonstrate that circulating MG53 protein can enter the cornea and exert positive healing effects. However, the mechanism by which extracellular MG53 enters cells and mediates its biological functions remain unclear. To identify potential membrane receptors responsible for MG53 binding, we employed a Membrane Proteome Array (MPA) platform. The MPA is a high‐throughput flow cytometry–based screening system encompassing 5380 distinct human membrane proteins (covering 94% of all human membrane proteins), including most single‐pass, multi‐pass, and GPI‐anchored proteins (including GPCRs, ion channels, and transporters) expressed in live cells under native conformations. Using this approach, we screened rhMG53 for its ability to bind to membrane proteins expressed in HEK‐293T cells under optimized assay conditions (1.25 µg/mL ligand concentration).

**FIGURE 7:**
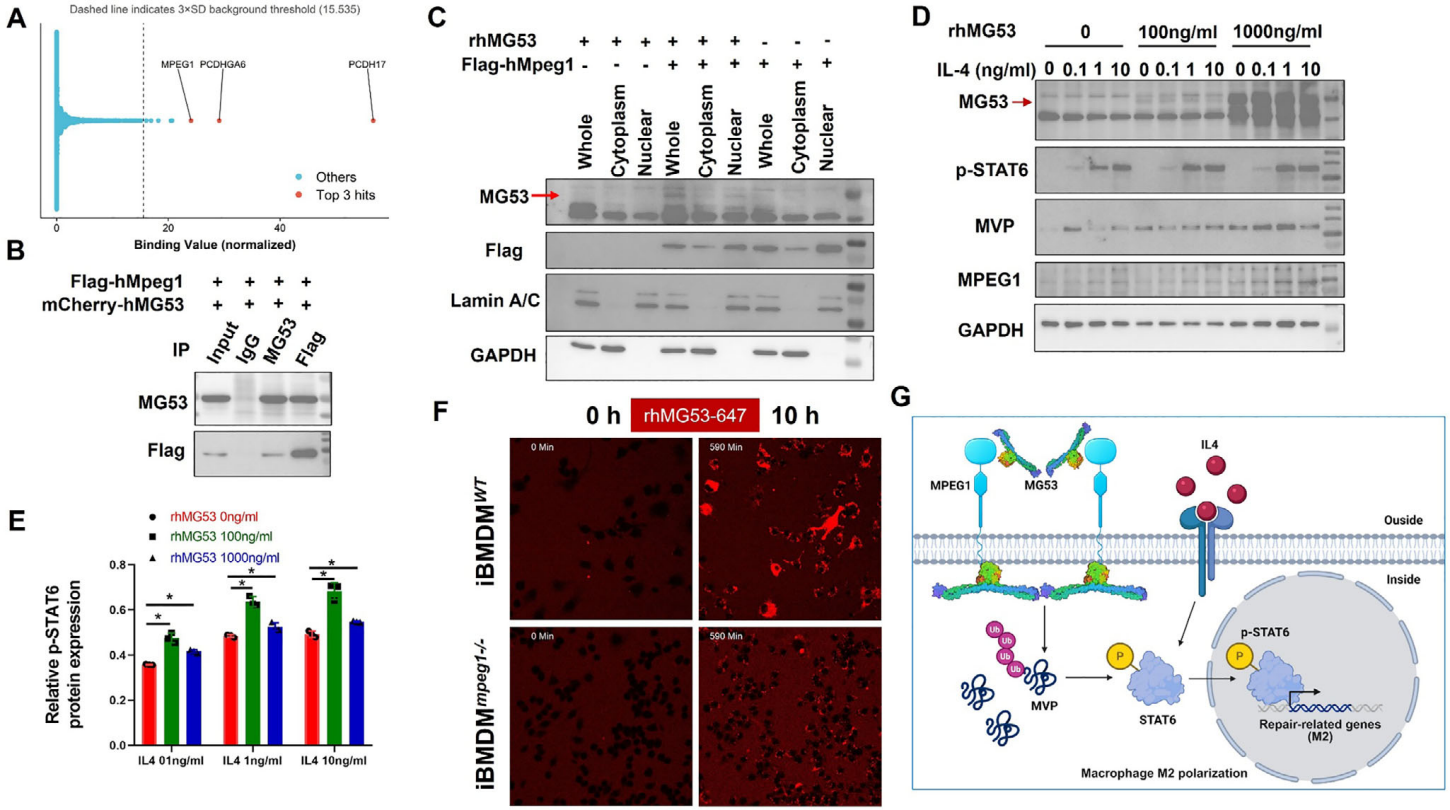
MG53 interacts with MPEG1 to regulate MVP–STAT6 signaling and promote macrophage M2 polarization. (A) Membrane Proteome Array (MPA) screening identified MPEG1 as one of the top binding candidates for MG53, along with PCDHGA6 and PCDH17. (B) Co‐immunoprecipitation showing the interaction between Flag‐tagged human MPEG1 and mCherry‐MG53 in HEK293T cells. (C) Western blot analysis of cytoplasmic and nuclear fractions in HEK293T cells overexpressing Flag‐hMPEG1 and treated with recombinant human MG53 (rhMG53, 40 ng/mL). Overexpression of MPEG1 facilitated cellular entry of exogenous MG53, as detected in both cytoplasmic and nuclear fractions. Lamin A/C and GAPDH served as nuclear and cytoplasmic markers, respectively. (D) Western blot analysis of IL‐4–induced signaling in BMDMs treated with different doses of rhMG53. MG53 enhanced IL‐4–induced STAT6 phosphorylation. (E) Quantification of p‐STAT6 levels normalized to total protein expression. Data are presented as mean ± SD from three independent experiments. ^*^ *p* < 0.05. (F) Representative fluorescence images of rhMG53‐647 uptake in WT and *Mpeg1‐/‐* iBMDMs at 0 and 10 h. MPEG1 deficiency markedly reduced rhMG53 internalization. (G) Proposed model illustrating that MG53 interacts with membrane protein MPEG1 to regulate MVP–STAT6 signaling, promoting STAT6 phosphorylation, nuclear translocation, and activation of repair‐related M2 macrophage genes.

As shown in Figure 7A, the MPA screen revealed several candidate membrane proteins with binding values above the 3×SD background threshold, among which MPEG1, PCDHGA6, and PCDH17 ranked among the top three hits. Notably, MPEG1, coded by *macrophage expressed gene 1*, is a macrophage‐specific membrane protein, making it a particularly interesting candidate receptor mediating MG53–macrophage interactions.

To validate this finding, we performed co‐immunoprecipitation assays in HEK‐293T cells co‐expressing Flag‐tagged human MPEG1 and mCherry‐tagged MG53. MG53 was efficiently pulled down with the Flag antibody, confirming a specific interaction between MG53 and MPEG1 (Figure 7B). Furthermore, subcellular fractionation analysis revealed that rhMG53 could enter the cells and translocate into the nucleus when MPEG1 was overexpressed (Figure 7C).

We further treated immortalized BMDMs with various concentrations of rhMG53 and IL‐4. The results showed that MG53 protein levels increased in a dose‐dependent manner with rhMG53 treatment, confirming effective uptake of the recombinant protein. IL‐4 stimulation induced phosphorylation of STAT6, and this activation was further enhanced by rhMG53, suggesting that MG53 augments IL‐4–STAT6 signaling (Figure 7D,E).

To further determine whether MPEG1 mediates the cellular uptake of MG53 in macrophages, we examined the internalization of fluorescently labeled rhMG53 (rhMG53‐647) in WT and *Mpeg1^−/−^* iBMDMs. As shown in Figure 7F, WT iBMDMs exhibited robust intracellular accumulation of rhMG53‐647 after 10 h of incubation (Movie S1), whereas *Mpeg1* ^−/−^ iBMDMs displayed markedly reduced fluorescence signals (Movie S2). These findings indicate that MPEG1 is required for efficient uptake of exogenous MG53 in macrophages, thereby facilitating downstream STAT6 activation.

We then performed rescue experiments in WT, *MPEG1^−/−^* and *MVP^−/−^* cells. As shown in Figure S10A,B, IL‐4 induced STAT6 phosphorylation to a comparable extent in WT and *MPEG1^−/−^* cells in the absence of rhMG53, indicating that MPEG1 deficiency does not markedly affect baseline IL‐4–STAT6 activation. However, upon rhMG53 treatment, IL‐4–induced STAT6 phosphorylation was significantly enhanced in WT cells but not in *MPEG1^−/−^* cells, demonstrating that MPEG1 is specifically required for the MG53‐dependent augmentation of STAT6 signaling. Consistent with this, the rhMG53‐mediated enhancement of STAT6 phosphorylation was markedly attenuated in *MVP^−/−^* cells, indicating that MVP is required for MG53‐driven STAT6 activation. Reconstitution of *MVP^−/−^* cells with WT MVP restored the ability of rhMG53 to enhance STAT6 phosphorylation, whereas expression of the ubiquitination‐defective mutant *MVP^K747R^* failed to rescue STAT6 activation. These results demonstrate that K747‐dependent ubiquitination of MVP is essential for MG53‐driven STAT6 signaling.

Together, these data suggest that MPEG1 functions as a potential cell‐surface receptor for MG53 in macrophages, facilitating MG53 internalization and downstream signaling activation for macrophage polarization. A working model is summarized in Figure 7G, illustrating how MG53 binding to MPEG1 may trigger MVP‐mediated K63‐linked ubiquitination and STAT6 activation, thereby promoting macrophage M2 polarization, nerve regeneration, and corneal wound healing.

## 3 Discussion

Corneal wound healing is a multifaceted process that requires precise coordination between epithelial regeneration, stromal remodeling, nerve regeneration, and vascular regulation. Among these, corneal nerve regeneration represents a particularly intricate event because it requires not only axonal outgrowth but also dynamic crosstalk between neurons and immune cells. Although various molecular mediators have been implicated in these processes, how immune regulation is mechanistically coupled to corneal nerve repair remains an unresolved question.

Under physiological conditions, resident macrophages in the cornea maintain a close anatomical relationship with sensory nerves. Seyed‐Razavi et al. first described a population of nerve‐associated macrophages (NAMs) that form intimate physical contacts with stromal nerve trunks [50]. These cells rapidly detach from the nerves within hours of epithelial injury and reestablish their association during the recovery phase, a process dependent on Cx3cr1 signaling. This observation indicates that macrophages are not passive bystanders but active sentinels capable of sensing neuronal stress and initiating a neuroimmune response. Such spatial coupling likely enables macrophages to rapidly detect and respond to axonal damage, thereby promoting corneal homeostasis.

Our findings build on this concept by demonstrating that macrophages are critical for corneal nerve regeneration and that MG53 acts as a central modulator of this process. Following alkali‐induced injury, macrophages rapidly accumulate at the limbus and around degenerating nerve fibers. Within 6 h, CD68^+^ macrophages begin to engulf β3‐tubulin–positive neuronal fragments (Figure 1D), suggesting their active role in debris clearance. Macrophage depletion using clodronate liposomes severely impaired both epithelial closure and nerve regeneration, confirming macrophages dual reparative and neuroprotective functions (Figure 2).

MG53 not only enhances epithelial and stromal healing, as previously established, but also promotes M2‐type macrophage polarization, a phenotype associated with tissue repair and axonal regeneration. Mechanistically, MG53 binds to MPEG1 on macrophage surface, enters macrophages and interacts with major vault protein (MVP), promoting its K63‐linked ubiquitination at lysine 747, which facilitates STAT6 nuclear translocation and M2 gene activation. This expands MG53's known E3 ligase repertoire beyond its canonical degradative role, highlighting MG53's ability to drive non‐degradative ubiquitination signaling that drives macrophage transcriptional programs toward a reparative state.

The early activation of macrophages following nerve injury is a conserved feature across models. Lee et al. demonstrated that corneal nerve transection induces a transient surge of NGF within ~3 h post‐injury, coinciding with the recruitment of Ly6C^+^ monocytes that rapidly differentiate into M2‐like macrophages [51]. This response precedes neutrophil infiltration and is accompanied by the temporary loss of resident F4/80^+^ macrophages, suggesting their mobilization to the injury site. NGF not only promotes macrophage polarization but also activates anti‐inflammatory and regenerative transcriptional programs.

Recent studies have clarified that corneal macrophages are not homogeneous. Liu et al. identified two distinct macrophage subsets: CCR2^+^ monocyte‐derived macrophages that dominate early inflammation and CCR2^−^ tissue‐resident macrophages that exert anti‐inflammatory and reparative functions [52, 53]. CCR2^+^ macrophages exhibit an M1‐like phenotype, producing IL‐1β and TNF‐α to initiate clearance, while CCR2^−^ macrophages express IL‐10 and Arg1 to suppress inflammation and promote resolution. Loss of either subset disrupts healing; CCR2^+^ depletion impairs debris clearance, whereas CCR2^−^ depletion leads to uncontrolled inflammation and delayed repair. This duality underscores that successful corneal regeneration depends on precise temporal coordination between inflammatory and reparative macrophage programs.

Beyond their polarization states, macrophages and other myeloid cells possess intrinsic feedback mechanisms that limit collateral damage. Lee et al. showed that CD11b^+^ myeloid cells protect corneal nerves from sterile injury through negative feedback regulation of the TLR2–IL‐6 axis [54]. In their model, loss of myeloid cells or IL‐6 dysregulation caused sustained inflammation and aggravated neurodegeneration. This demonstrates that myeloid cells not only initiate inflammation but also actively engage in its resolution by restraining TLR‐driven cytokine production. In addition to immune cells modulating nerves, nerves also regulate immune activity. Liu et al. revealed that TRPV1^+^ sensory nerves release the neuropeptides CGRP and SST, which signal through RAMP1 and SSTR5 to orchestrate macrophage behavior [52]. CGRP–RAMP1 signaling suppresses pro‐inflammatory CCR2^+^ macrophages and neutrophils, while SST–SSTR5 signaling enhances IL‐10 production in CCR2^−^ macrophages. This bidirectional communication ensures immune homeostasis and efficient repair. The disruption of this circuit, as seen in diabetic neuropathy, leads to prolonged inflammation and delayed healing.

Integrating these findings, we propose that MG53 serves as a coordinator of neuro–immune repair. Circulating MG53 binds to MPEG1 on macrophages, leading to MVP ubiquitination and STAT6‐dependent M2 polarization, which accelerates clearance of degenerating axons and promotes nerve regrowth. In parallel, this mechanism interfaces with existing neuro–immune circuits, such as NGF‐driven macrophage recruitment and TRPV1‐mediated neuropeptide signaling, to reinforce corneal regeneration.

From a translational perspective, MG53 modRNA delivery, via direct corneal injection or systemic administration of secretory tPA‐MG53, produced robust therapeutic benefit. MG53 modRNA accelerated epithelial closure, suppressed fibrosis and neovascularization, and enhanced corneal nerve regeneration, with sustained MG53 expression for up to 7 days after a single injection. This long‐lasting, cell‐intrinsic activation of reparative macrophage signaling suggests that MG53‐based therapies could offer a durable and non‐invasive approach for treating ocular surface injuries and potentially other neuroimmune repair contexts such as myocardial infarction or peripheral neuropathy.

Despite the promising findings, there are questions that remain to be elucidated in future studies. First, MPA identified other potential receptors, such as PCDH17 and PCDHGA6. Interestingly, PCDH17 and PCDHGA6 have been shown to play an important role in neuron functions, controlling nerve cell death, and even serving as neuronal markers [55, 56]. Therefore, it is possible that MG53 might protect sensory nerve via directly binding to PCDH17 and PCDHGA6. Second, previous study showed that MG53 plays a role in infection induced macrophage activation via intracellular calcium regulation [57], thus, intracellular calcium signal might also contribute to alkali injury‐induced sterile inflammation and nerve regeneration. Third, we have previously shown that neutrophils plays a role in the early phase of alkali‐induced corneal wound healing [58]; future studies will focus on the potential role of MG53 in other types of immune cells, including neutrophils, T cells, and mast cells.

Together, our findings delineate a multilayered neuro–immune repair network in which macrophages act as both effectors and regulators of corneal nerve regeneration. MG53 emerges as a molecular bridge linking extracellular membrane repair with intracellular transcriptional programming, aligning immune activation, inflammation resolution, and axonal regeneration within a unified framework. By integrating structural coupling [50], subset‐specific dynamics [53], early innate responses [51], and sensory feedback regulation [52], this study positions MG53–MPEG1–MVP–STAT6 signaling as a pivotal axis that sustains the delicate balance between immunity and regeneration in the injured cornea.

Despite the promising findings, several limitations warrant further investigation. First, Although MG53 modRNA and tPA‐MG53 delivery demonstrated therapeutic benefit, long‐term safety, pharmacokinetics, immune responses, and potential off‐target effects remain to be systematically evaluated. Second, while MG53 is well established as a muscle‐enriched protein functioning as a circulating myokine, the present study does not include cell type–specific genetic manipulation to definitively distinguish the relative contribution of macrophages vs. other cell populations. While our work focuses on M2 polarization, it does not fully address the heterogeneity of corneal macrophages. Distinct subsets, including CCR2^+^ infiltrating monocyte‐derived macrophages and CCR2^−^ tissue‐resident macrophages, may respond differently to MG53 signaling. Third, recent structural studies have shown that TRIM72 (MG53) undergoes higher‐order assembly on negatively charged membranes, which enhances RING dimerization and E3 ubiquitin ligase activity [18]. In this context, whether MPEG1‐mediated MG53 recruitment to macrophage membranes potentiates its ubiquitination activity through membrane localization remains to be determined. Future work dissecting the spatial regulation of MG53 E3 activity downstream of MPEG1 will further clarify this mechanism.

## 4 Materials and Methods

### 4.1 Animals

Wild‐type (WT) C57BL/6J mice, MG53 global knockout (*mg53‐/‐*) mice, and transgenic mice with elevated MG53 in circulation (tPA‐MG53) were used in this study. All animal procedures were approved by the Institutional Animal Care and Use Committee of The Ohio State University (IACUC #2016A00000017‐R3) and conformed to the ARVO Statement for the Use of Animals in Ophthalmic and Vision Research. Mice were maintained under a 12‐h light/dark cycle with free access to food and water.

### 4.2 Corneal Alkali Burn Injury Model

Corneal injury was induced by applying a circular piece of filter paper (2 mm diameter) soaked in 2 uL 1 m NaOH to the central cornea for 30 sec, followed by thorough rinsing with sterile saline. Topical antibiotic ointment was applied immediately after injury. Corneal tissues were collected at specified time points (0, 1, 3, 7 or 10 days post‐injury) for immunostaining or Western blot analysis. For macrophage depletion experiments, mice were injected intravenously with 10 µl/g (5 mg/ml) clodronate liposomes  (or PBS liposomes as control) 48 h before injury, immediately before injury, and again 48 h after injury. For PM43I treatment, PM43I was dissolved in DMSO to prepare a 10 mm stock solution. Mice received intraperitoneal (i.p.) injections of 10 µL PM43I once daily, starting one day prior to corneal injury and continuing until euthanasia. Control animals were administered an equivalent volume of DMSO following the same schedule.

### 4.3 Clinical Evaluation

The clinical opacity scores and clinical vascularization scores were determined by single observer, masked to the animal grouping, using a modified Hackett–McDonald scoring system. The evaluations conducted immediately following cornea injury and subsequently every 24 h. For specific scoring criteria, please refer to our previous publication [59]. Size of the cornea wound was verified using fluorescein.

The mouse eye images in the aforementioned examination were captured using the Leica THUNDER Imaging System. Images of wound fluorescein were quantified by using ImageJ software.

### 4.4 Immunofluorescence Staining and Imaging

Dissected eyes from mice were fixed in 4% paraformaldehyde (PFA) for 24 to 48 h at 4°C. The eyes were processed as follows: 2 h 50% ethanol, 1.5 h 70% ethanol, 1 h 80% ethanol, 1 h 90% ethanol, 30 min 95% ethanol, 30 min 95% ethanol, 15 min 100% ethanol, 15 min 100% ethanol, 15 min xylene, 15 min xylene, 30 min paraffin wax, 1 h paraffin wax, 1 h paraffin wax. After embedding, 5 µm thick paraffin sections were cut for IF staining. The procedures for IF staining were: deparaffinise/hydrate sections: xylene I: 7 min, xylene II: 7 min, 100% ethanol I: 5 min, 100% ethanol II: 5 min, 95% ethanol: 3 min, 70% ethanol: 3 min, 50% ethanol: 3 min, ddH2O I: 3 min, ddH2O II: 3 min. Antigen retrieval was performed using tris‐EDTA buffer (10 mM Tris, 1 mM EDTA, pH *9.0*), microwaved for 15 min. Slides were then washed in PBS 3 times/3 min then blocked in 3% bovine serum albumin (BSA) in phosphate‐buffered saline with tween 20 (PBST) for 1 h at room temperature. Blocking solution was removed and slides incubated with primary antibody against αSMA (Sigma, cat# A5228, 1:1000 dilution) diluted in tris‐buffered saline with tween 20 (TBST) with 1% BSA overnight at 4°C. Slides were then washed in PBST three times for 5 min each. Fluorescently labelled secondary antibody (Thermo, cat#A21202, Donkey anti‐Mouse IgG, 488), diluted in TBST with 1% BSA was then added to each slide and incubated for 60 min at room temperature. Slides were then washed in PBST three times for 5 min each. Slides were mounted with coverslips and mounting medium containing DAPI. The immunofluorescence staining images were captured using the A1R laser confocal microscope from Nikon.

### 4.5 Corneal Flat‐Mount Staining

Corneal flat‐mount staining was performed as previously described, with minor modifications (adapted from STAR Protocols) [60]. Briefly, eyes were washed with cold PBS immediately after collection and fixed in 1.5% PFA for 1 h at room temperature (RT). After washing with PBS, corneas were dissected and washed five times (5 min each, RT). Samples were permeabilized in 1% Triton X‐100 (in PBS) for 1 h at RT, followed by blocking in 0.3% Triton X‐100, 0.1% Tween‐20, and 20% BSA (in PBS) for 30 min. Corneas were then incubated with primary antibodies against β3‐tublin (Thermo Scientific, cat#PA5‐85639), CD31 (BD Biosciences, cat#550274), MPO (R&D Systems, cat#AF3667), CD206 (BioLegend, Alexa Fluor 647, cat#141712), CD80 (Thermo Fisher, cat#16‐0801‐82), CD11b (BioLegend, cat#101262), CD192 (BioLegend, cat#150605), CD68 (Bio‐Rad antibodies, cat#MCA1957) 1:200 for 2 h at RT and overnight at 4°C. After five washes with 0.1% Tween‐20 in PBS, tissues were incubated with secondary antibodies (Thermo Scientific, cat#A21202, cat#A31572, cat#A21447, cat#A21434) (1:300, diluted in blocking buffer) for 2 h at RT, followed by another five washes (10 min each). Corneas were flat‐mounted on slides and dried at 4°C for 12–24 h before imaging. The immunofluorescence staining images were captured using the A1 laser confocal microscope or Fi3 fluorescent microscope from Nikon, and nerve density and neovascularization ratios were quantified using ImageJ. The neovascularization ratio was calculated by dividing the area occupied by blood vessels and the total corneal area. The nerve area ratio was calculated similarly by dividing the nerve‐stained area by the total corneal area.

### 4.6 Isolation and Polarization of Bone Marrow–Derived Macrophages (BMDMs)

Bone marrow cells were isolated from the femurs and tibias of WT, *mg53‐/‐*, and tPA‐MG53 mice. Cells were cultured in DMEM supplemented with L929 cell–conditioned medium as a source of M‐CSF for 7 days to generate BMDMs. M2 polarization was induced with 20 ng/mL IL‐4 for 24 h. The expression of M2‐associated genes (Arg1, Fizz1, Ym1/2, and IRF4) was assessed by qRT‐PCR and Western blotting.

### 4.7 Co‐Immunoprecipitation (Co‐IP) and Western Blot Analysis

Cells or tissue lysates were prepared using RIPA lysis buffer containing protease and phosphatase inhibitors. For CO‐IP, lysates were incubated with specific antibodies (1:100) against MG53 (homemade, cat#914), MVP (Thermo Scientific, cat#PA5‐115604), Flag (Sigma, cat#F1804), Ubc13 (Thermo Scientific, cat#37‐1100) overnight at 4°C, followed by incubation with Protein A/G agarose beads (Invirtrogen, cat#10003d, cat#10001D). After washing, immunoprecipitates were analyzed by SDS‐PAGE and immunoblotting. For ubiquitination assays, K48‐ (Cell Signaling Technology, cat#12805S) and K63‐linked (Cell Signaling Technology, cat#12930S) ubiquitin–specific antibodies were used. For Western blot, proteins were separated by using SDS‐PAGE gels, then transferred to PVDF membranes. Membranes were blocked and incubated with primary antibodies against MG53 (homemade, cat#914), MVP (Thermo Scientific, cat#PA5‐115604), Flag (Sigma, cat#F1804), Ubc13 (Thermo Scientific, cat#37‐1100), STAT6 (Thermo Scientific, cat#MA5‐15659), p‐STAT6 (Thermo Scientific, cat#700247), GAPDH (Cell Signaling Technology, cat#2118S), LaminA/C (Abcam, cat#ab108595) overnight. Following extensive washing, membranes were incubated with species‐specific secondary antibodies and protein bands were visualized using chemiluminescence and quantified by ImageJ.

### 4.8 Mass Spectrometry Analysis

Co‐IP products were separated by SDS‐PAGE and visualized with Coomassie blue staining. All mass spectrometry (MS) experiments were performed at the Mass Spectrometry & Proteomics (MSP) Facility, part of the Campus Chemical Instrument Center, The Ohio State University (Columbus, OH). Samples were submitted according to their sample submission guidelines and processed using the facility's standard protocols for protein identification/quantification.

### 4.9 Membrane Proteome Array (MPA) Screening

Membrane Proteome Array (MPA) screening was performed at Integral Molecular, Inc. (Philadelphia, PA) using the company's proprietary high‐throughput flow cytometry platform.

The MPA screened 5380 distinct human membrane protein clones, covering over 94% of all known single‐pass, multi‐pass, and GPI‐anchored membrane proteins, including GPCRs, ion channels, and transporters. Each clone was transiently expressed in live HEK‐293T cells in individual wells of 384‐well plates and arrayed in duplicate in a matrix format to enable deconvolution of binding events.

Recombinant human MG53 (rhMG53) was tested at 1.25 µg/mL (in 10% goat‐serum blocking buffer) under the optimized screening conditions provided by Integral Molecular. Ligand binding was detected by high‐throughput immunofluorescence flow cytometry using an Intellicyt iQue system.

Binding signals exceeding three standard deviations above background in both intersecting matrix wells were considered positive hits. Identified candidates were then validated by secondary titration assays performed under the same flow cytometric conditions, where targets were confirmed if the mean fluorescence intensity (MFI) was at least twofold above background at two consecutive ligand concentrations.

### 4.10 Plasmid Construction and Site‐Directed Mutagenesis

The wild‐type MVP plasmid pEGFP‐C2‐MVP (Addgene plasmid #204543) was used as the backbone for mutagenesis. Site‐directed point mutations were introduced into the MVP coding sequence using Q5 Site‐Directed Mutagenesis Kit (NEB, E0554S), with mutation‐specific primers. All mutated constructs were verified by Sanger sequencing.

The human MPEG1 expression plasmid (Cat#: HG13993‐NF) was obtained from Sino Biological Inc. (Beijing, China). This construct contains the full‐length human MPEG1 cDNA (NM_001039396.1) cloned into the pCMV3‐SP‐N‐FLAG vector, encoding an N‐terminal FLAG tag under control of the CMV promoter. The open reading frame size is 2166 bp, and the insert sequence is identical to the corresponding NCBI reference sequence.

### 4.11 Cell Culture, Transfection, and rhMG53 Treatment

HEK‐293T cells were maintained in Dulbecco's modified Eagle medium (DMEM; Gibco) supplemented with 10% fetal bovine serum (FBS) and 1% penicillin/streptomycin at 37°C in a humidified atmosphere containing 5% CO_2_. Transient transfections were carried out using Lipofectamine 3000 (Thermo Fisher Scientific) according to the manufacturer's instructions. Nuclear–cytoplasmic fractionation was performed, with minor modifications, based on a previously published protocol [61]. Briefly, cells were harvested 24–36 h after transfection, and the cell pellet was resuspended in an appropriate volume of 0.1% NP‐40 lysis buffer supplemented with protease and phosphatase inhibitors (PI and PPI). The lysate was divided equally into two portions: one half was mixed with one‐third volume of RIPA buffer and designated as the whole‐cell lysate. The remaining half was centrifuged at 15 000 rpm for 5 min at 4°C to separate the supernatant (cytoplasmic fraction) and pellet (nuclear fraction). The cytoplasmic fraction was collected and combined with one‐third volume of RIPA buffer. The nuclear pellet was washed twice with 0.1% NP‐40 buffer containing PI and PPI, then resuspended in the same volume of RIPA buffer used for the whole‐cell and cytoplasmic lysates. Nuclear samples were sonicated briefly and centrifuged to remove debris. All lysates were mixed with 6× loading buffer and boiled for 5 min before immunoblot analysis.

The Immortalized macrophage was kindly provided by Dr. Haitao Wen from the Ohio State University. Primary bone marrow–derived macrophages (BMDMs) were isolated from mouse femurs and tibias. Bone marrow cells were flushed with PBS containing 2% FBS and cultured in BMM growth medium (DMEM supplemented with 20% heat‐inactivated FBS, 30% L929 cell–conditioned medium, and penicillin/streptomycin). Cells were maintained for 5–6 days to allow differentiation into M0 macrophages. For M2 polarization, differentiated M0 BMDMs were treated with recombinant murine IL‐4 at the indicated concentration for 24 h prior to analysis.

For the transfection of modified RNA, BMDMs were cultured in 6‐well plates and transfected with 1 µg of modified RNA per well using Lipofectamine 3000 (Thermo Fisher Scientific) following the manufacturer's instructions. After 24–36 h of transfection, cells were collected and subjected to subsequent analyses or treatments as indicated.

Plasmid transfection in iBMDMs was performed using the Neon Electroporation System (Thermo Fisher Scientific) according to the manufacturer's instructions. For generation of knockout cell lines, electroporation was carried out under the following optimized conditions: 1400 V, 10 ms pulse width, and 3 pulses, to achieve maximal transfection efficiency. At 24–36 h post‐transfection, cells were subjected to selection with puromycin (10 µg/mL), followed by isolation and expansion of single‐cell clones. For overexpression experiments, electroporation parameters were adjusted to 1400 V, 10 ms, and 2 pulses to improve cell viability. The CRISPR plasmid PX459 V2.0‐eSpCas9(1.1) was obtained from Addgene (cat. #108292, Watertown, MA, USA). Guide RNAs (gRNAs) were designed using the CRISPR design tool available at crisprscan.org. Detailed knockout procedures were performed as previously described in our published work [62].

### 4.12 Modified RNA (modRNA) Preparation and Delivery

Chemically modified RNAs encoding MG53, HA–tPA–MG53, or GFP were synthesized using the Takara IVTpro mRNA Synthesis System (Cat. #6141, BspQI version), which contains a T7 promoter and is optimized for co‐transcriptional capping using CleanCap Reagent AG‐3′OMe (TriLink, Cat. #N‐7413). During in vitro transcription (IVT), N1‐methylpseudouridine‐5′‐triphosphate (TriLink, Cat. #N‐1081) was substituted for UTP to minimize innate immune activation and improve mRNA stability. The remaining ribonucleotides (ATP, CTP, and GTP; provided in the Takara kit, Cat. #6144) were used at equimolar concentrations. Transcription reactions were incubated at 37°C for 2 h with T7 enzyme mix, followed by DNase I digestion to remove plasmid DNA. mRNA was purified using LiCl precipitation to remove enzymes and unincorporated nucleotides. RNA concentration and purity were assessed using NanoDrop spectrophotometry and agarose gel electrophoresis. For local tissue delivery, 6 µg of purified modRNA was complexed with in vivo‐jetRNA+ (Polyplus, Cat. #101000122) using a ratio of 1:2 (µg modRNA: µL reagent) in mRNA buffer (included in the kit) to a final injection volume of 6 µL, and injected into the corneal stroma using a 33G Hamilton microsyringe, ensuring precise intrastromal localization. For systemic expression, 10 µg of HA–tPA–MG53 modRNA was injected intramuscularly into the quadriceps muscle. Protein expression profiles were evaluated at 1, 3, 7, and 10 days post‐injection by Western blotting of corneal tissue and serum samples.

### 4.13 RNA Extraction and Quantitative PCR

Total RNA was extracted from corneas or macrophages using TRIzol reagent. Reverse transcription was performed using a cDNA synthesis kit, followed by quantitative PCR with SYBR Green Master Mix. Gene expression levels were normalized to Gapdh and analyzed using the 2^–ΔΔCt method. The primers used are listed as following: mouse‐Ubc: Forward‐5′‐ccagtgttaccaccaagaag‐3′, Reverse‐5′‐acccaagaacaagcacaagg‐3′; mouse‐Arg1: Forward‐5′‐ gtgaagaacccacggtctgt‐3′, Reverse‐5′‐ctggttgtcaggggagtgtt‐3′; mouse‐Fizz1: Forward‐5′‐ tgctgggatgactgctactg‐3′, Reverse‐5′‐ctgggttctccacctcttca‐3′; mouse‐Ym1/2: Forward‐5′‐ gggcatacctttatcctgag‐3′, Reverse: Forward‐5′‐ccactgaagtcatccatgtc‐3′; mouse‐IRF4: Forward‐5′‐gagccaagcataaggtctgc‐3′,Reverse: Forward‐5′‐tttttcctctggccattgtc‐3′.

### 4.14 Statistical Analysis

All quantitative data are presented as mean ± SD or SEM. Colocalization analysis was performed using the Coloc 2 plugin in Fiji (ImageJ), and Pearson's correlation coefficient was calculated to quantify the degree of colocalization. Statistical analyses were performed using GraphPad Prism. Comparisons between two groups were made by unpaired Student's t‐test, while multiple‐group comparisons were analyzed by one‐way ANOVA with Tukey's post hoc test. The sample size (n) for each experiment used in statistical analyses is indicated in the corresponding figure legends. A p‐value < 0.05 was considered statistically significant.

## Conflicts of Interest

The authors declare no conflict of interest.

## Supporting information




**Supporting File 1**: advs75206‐sup‐0001‐SuppMat.docx.

**Supporting File 2**: advs75206‐sup‐0002‐MovieS1.avi.

**Supporting File 3**: advs75206‐sup‐0003‐MovieS2.avi.

## Data Availability

The data that support the findings of this study are available from the corresponding author upon reasonable request.
