# Supplementary material for: MG53 Coordinates Macrophage Polarization and Neuroimmune Coupling to Promote Corneal Nerve Regeneration via the MPEG1–MVP–STAT6 Axis
Source: Adv Sci (Weinh). 2026 Apr 10:e23002. Online ahead of print. doi: 10.1002/advs.202523002 (PMC13334635; doi:10.1002/advs.202523002)

**Supplementary Figure s1. Colocalization analysis of immune cells with corneal nerves after injury.**

(A) Representative regions of interest (ROIs) showing immunofluorescence staining of CD68 (green) or MPO (magenta) with β3-tubulin (red) at 3 h and 6 h after injury. For each cornea, three ROIs were selected for colocalization analysis.

(B) Quantification of colocalization between immune cell markers (CD68 or MPO) and β3-tubulin using Pearson’s correlation coefficient (R) calculated with the Coloc 2 plugin in Fiji. Each dot represents one mouse. Data are presented as mean ± SD (n = 3 mice per group). *p < 0.05.

**Supplementary Figure s2. Immunofluorescence staining of corneal flat mounts 24 hours after alkali burn.** Representative confocal images showing co-staining of macrophage markers (CD80, CD206, or CD163) with the neuronal marker β3-tubulin in the cornea 24 hours after alkali injury. Nuclei are counterstained with DAPI (blue).

**Supplementary Figure s3. Immunofluorescence staining of corneal flat mounts 24 hours after alkali burn.** Representative confocal images showing co-staining of β3-tubulin (red) with macrophage markers CD11b or CD192 (green) in corneal tissues 24 hours after alkali injury. Nuclei are counterstained with DAPI (blue).

**Supplementary Figure s4. Triple immunofluorescence staining of corneal flat mounts 24 hours after alkali burn.** Representative confocal images showing co-localization of CD68 (green), CD206 (red), and β3-tubulin (magenta) in the cornea 24 hours after alkali injury. Nuclei are counterstained with DAPI (blue).

**Supplementary Figure s5. MG53 promotes M2 macrophage polarization and corneal nerve regeneration after alkali injury.** (A) Representative corneal flat-mount immunofluorescence images showing CD206⁺ macrophages (cyan) and β3-tubulin⁺ nerve fibers (red) in *mg53-/-*, WT, and tPA-MG53 transgenic mice 2 days after alkali burn. Dashed circles indicate the central corneal area. (B) Quantification of CD206⁺ macrophages in the cornea. Data are presented as mean ± SD (n = 3 per each group). *p < 0.05. (C) Quantification of corneal nerve density. Data are presented as mean ± SD (n = 3 per each group). *p < 0.05.

**Supplementary Figure s6. PM43I treatment delays corneal wound healing after alkali injury. (A)** Representative bright-field and fluorescein staining images of corneas from control (DMSO) and PM43I-treated mice at Day 1 and Day 10 after injury. Fluorescein staining indicates epithelial defect areas. (n=5 per each group). **(B)** Quantification of corneal injury area ratio over time (Day 0–Day 10). PM43I-treated mice exhibited delayed epithelial wound closure compared with controls. (n=5 per each group). **(C)** Clinical opacity scores recorded at indicated time points. PM43I treatment resulted in persistently increased corneal opacity. **(D)** Neovascularization scores evaluated during the healing process. PM43I significantly increased corneal neovascularization at later time points. **(E)** Representative whole-mount corneal images showing nerve fibers (green) and neovascularization (red) in control and PM43I-treated groups. (n=5 per each group). **(F)** Quantification of corneal neovascularization area ratio. **(G)** Quantification of corneal nerve fiber density/area ratio. PM43I treatment significantly reduced corneal nerve regeneration. Data are presented as mean ± SD. *P < 0.05 versus control.

**Supplementary Figure s7. PM43I treatment reduces colocalization of macrophage and corneal nerves after alkali injury. (A)** Representative whole-mount corneal immunofluorescence images from WT mice treated with PM43I. Corneas were harvested at 3 h and 6 h post-injury. CD68 (green) marks macrophages and β3-tubulin (red) labels corneal nerve fibers. Insets show higher magnification views of the indicated regions. (n=3 per each group). **(B)** Representative whole-mount corneal immunofluorescence images from tPA-MG53 transgenic mice treated with PM43I. Corneas were collected at 3 h and 6 h post-injury. CD68 (green) and β3-tubulin (red) staining are shown. (n=3 per each group). **(C)** Representative high-magnification images showing regions of interest (ROIs) used for colocalization analysis between CD68-positive macrophages and β3-tubulin-positive nerve fibers at 3 h and 6 h post-injury. **(D)** Quantification of CD68/β3-tubulin colocalization expressed as Pearson’s correlation coefficient (R value). For each cornea, three independent ROIs were selected and averaged to obtain a single value per sample. Data represent the mean ± SD of three biological replicates (n = 3 mice per group). *p < 0.05; ns, not significant.

**Supplementary Figure s8. PM43I treatment impairs corneal nerve regeneration in both WT and tPA-MG53 mice. (A)** Representative β3-tubulin staining of corneal whole mounts at Day 7 post-injury in WT and tPA-MG53 mice treated with PM43I. (n = 5 per each group). **(B)** Quantification of nerve area ratio. tPA-MG53 mice exhibited significantly improved nerve preservation compared with WT mice under both untreated and PM43I-treated conditions. Untreated group, n = 3 per group; PM43I-treated group, n = 5 per group. Data are presented as mean ± SD. *P < 0.05.

**Supplementary Figure s9. Quantification of MG53 in corneas after injection of tPA-MG53 modRNA.**

**Supplementary Figure s10. (A)** Representative immunoblot analysis of IL-4–induced STAT6 phosphorylation in WT, *Mpeg1-/-* and *Mvp-/-* cells with or without recombinant human MG53 (rhMG53). Reconstitution of *Mvp-/-* cells with WT MVP or K747R mutant (MVP-K747R) was performed as indicated. Cells were stimulated with IL-4 (1 ng/ml) in the presence (1µg/ml) or absence of rhMG53. Protein levels of p-STAT6, MVP, MPEG1, MG53, and GAPDH are shown. **(B)** Quantification of relative p-STAT6 protein expression. Data are presented as mean ± SD from three independent experiments. *p < 0.05.

**Supplementary movies s1-2: Uptake of rhMG53 by iBMDM cells.** WT iBMDMs (movie s1) and *Mpeg1-/-* iBMDMs (movie s2) were incubated with Alexa-647 labeled rhMG53 protein and subjected to live cell confocal imaging. Live cell imaging was performed by Nikon A1 confocal system equipped with stage top environment control system to maintain the cells at 37 ^o^C, 5% CO_2_ and 95% humidity during the entire imaging process. Image recording rate was 10 mins/frame for 10 hours.


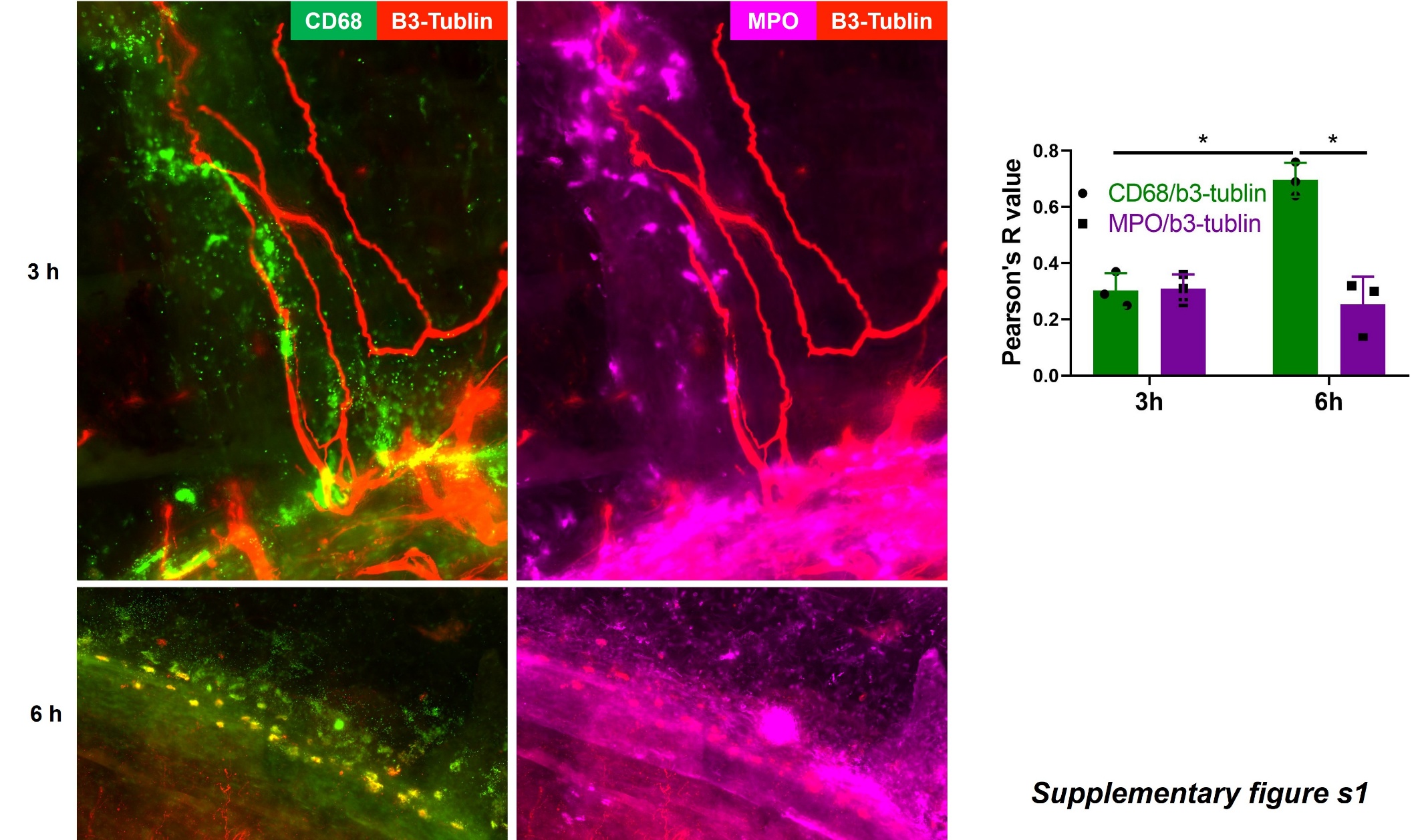


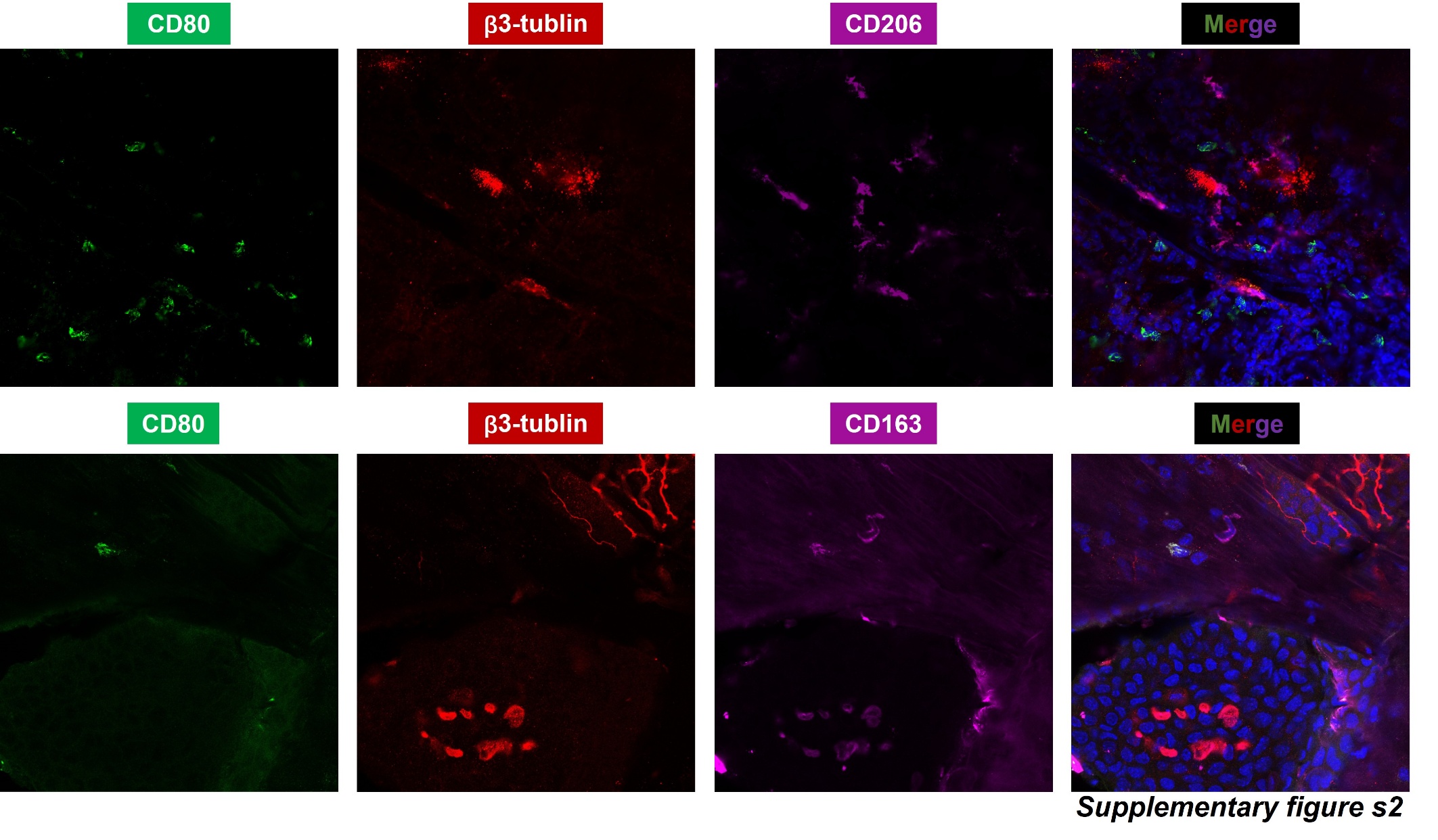


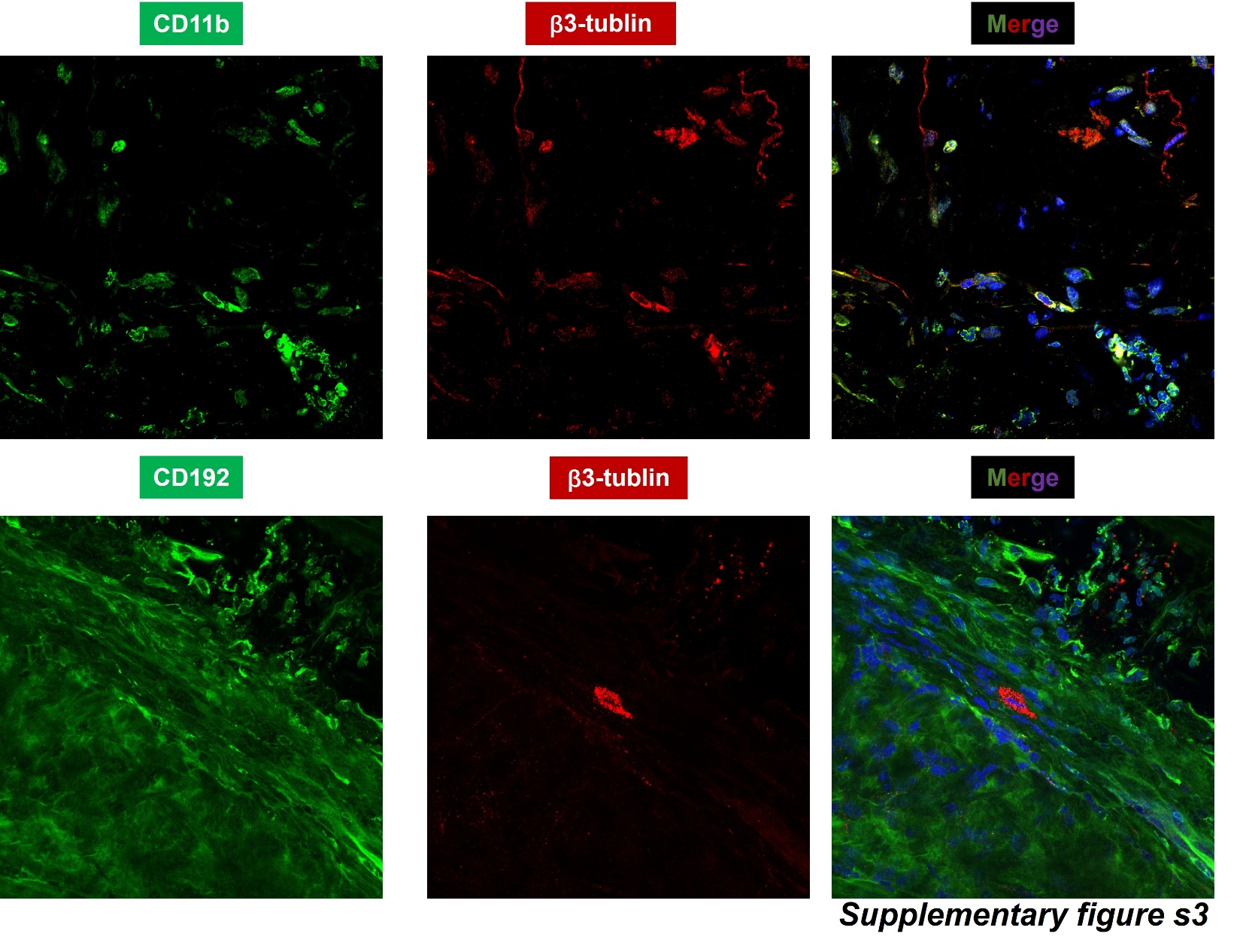


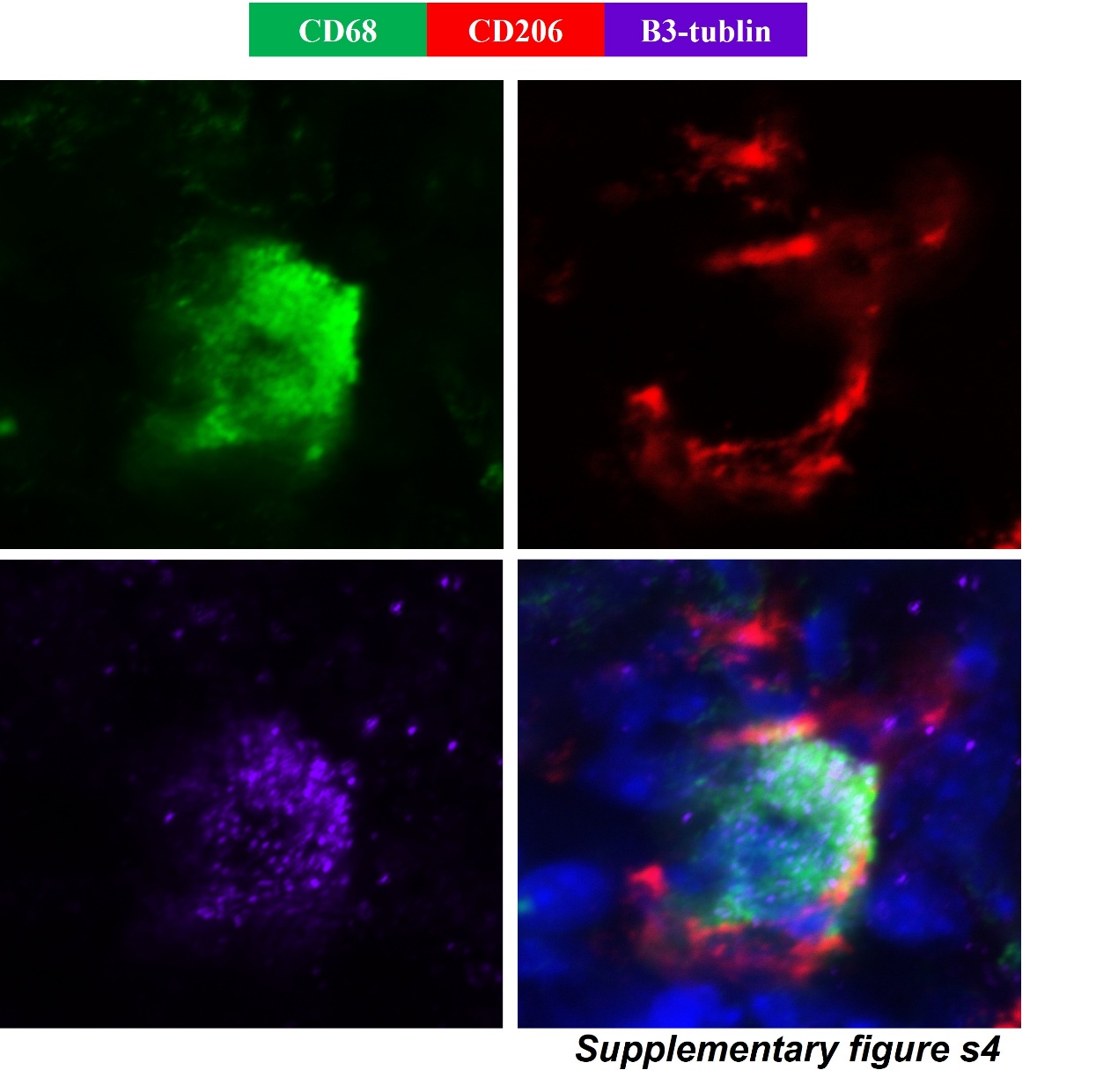


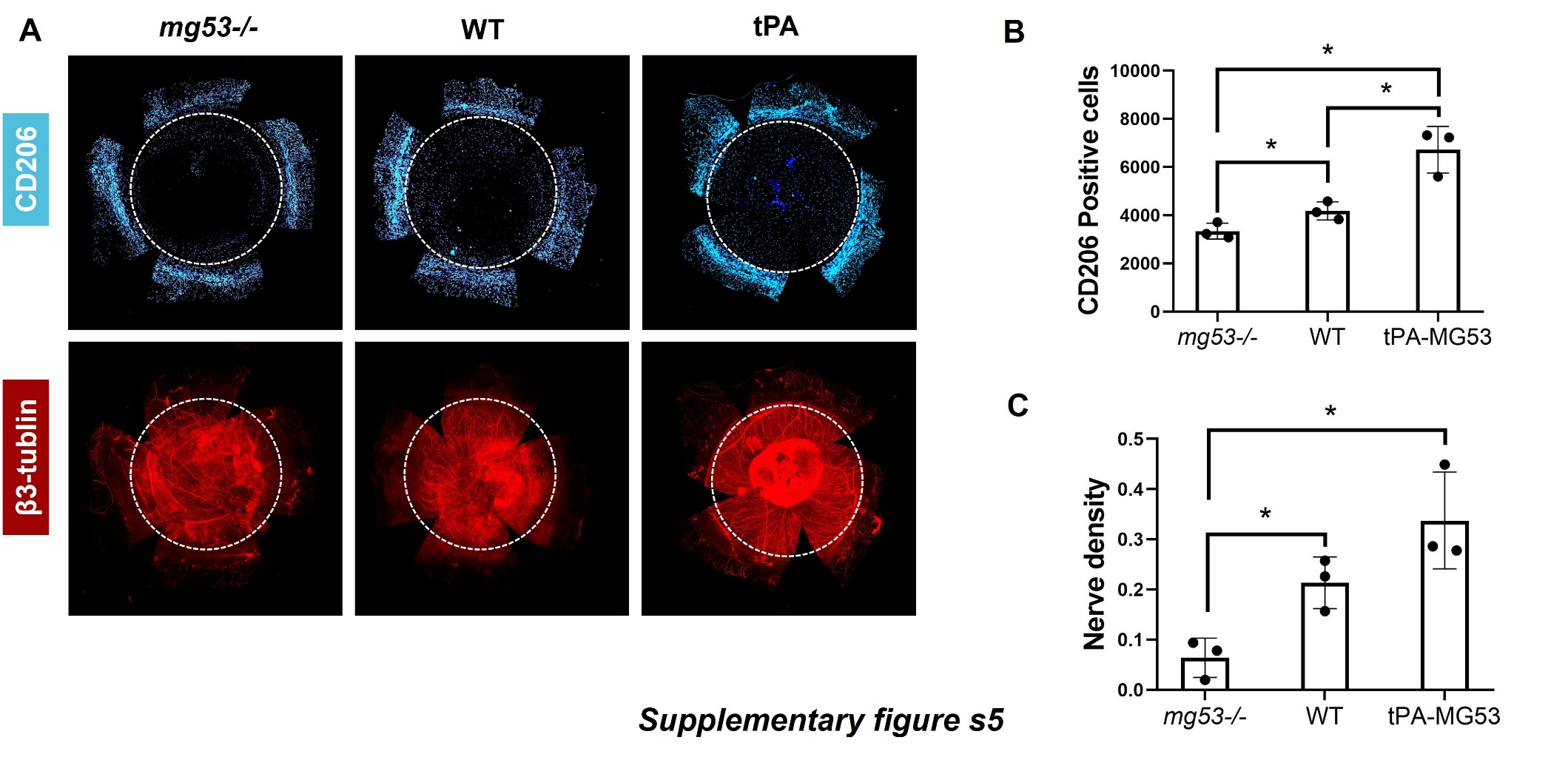


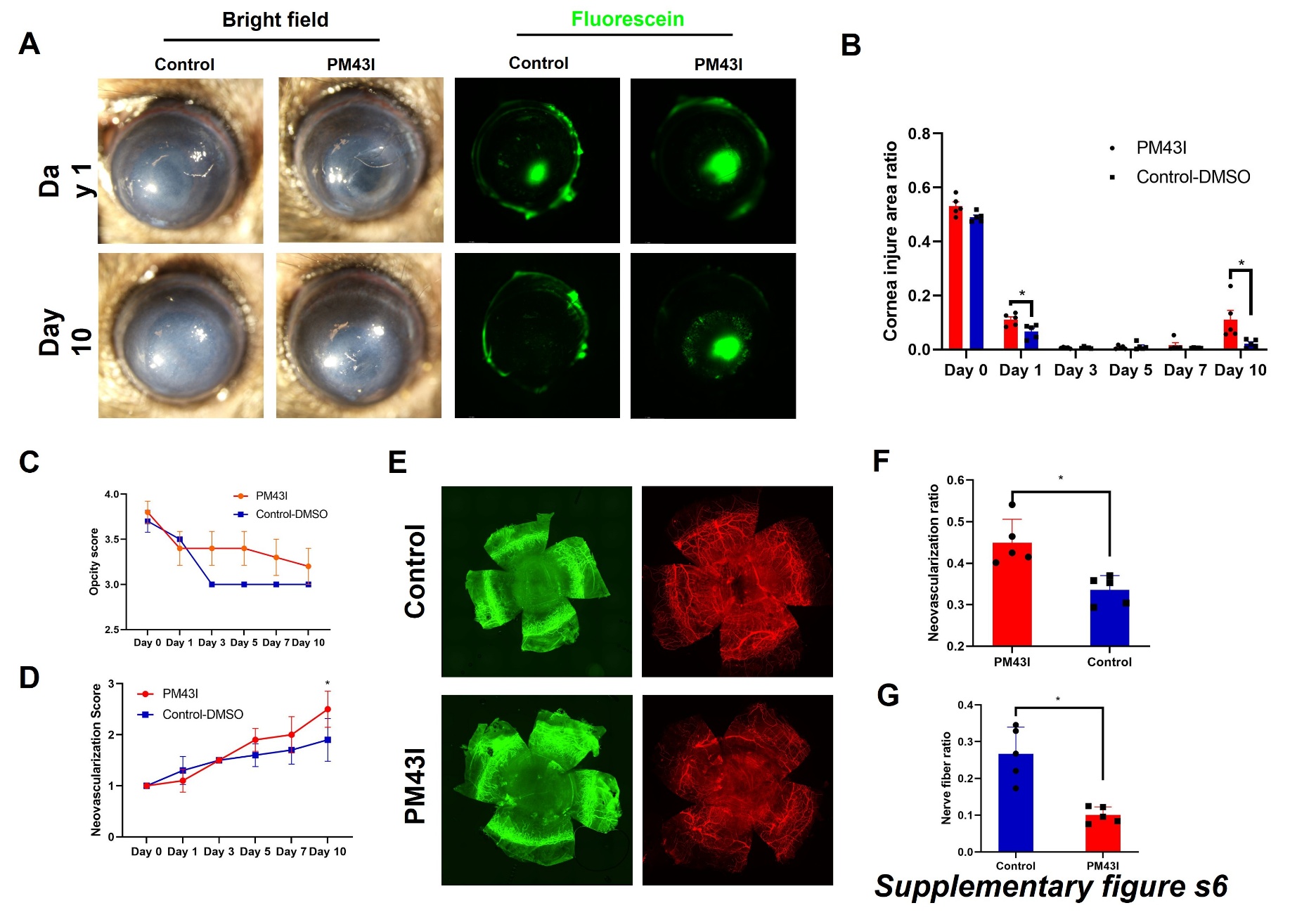


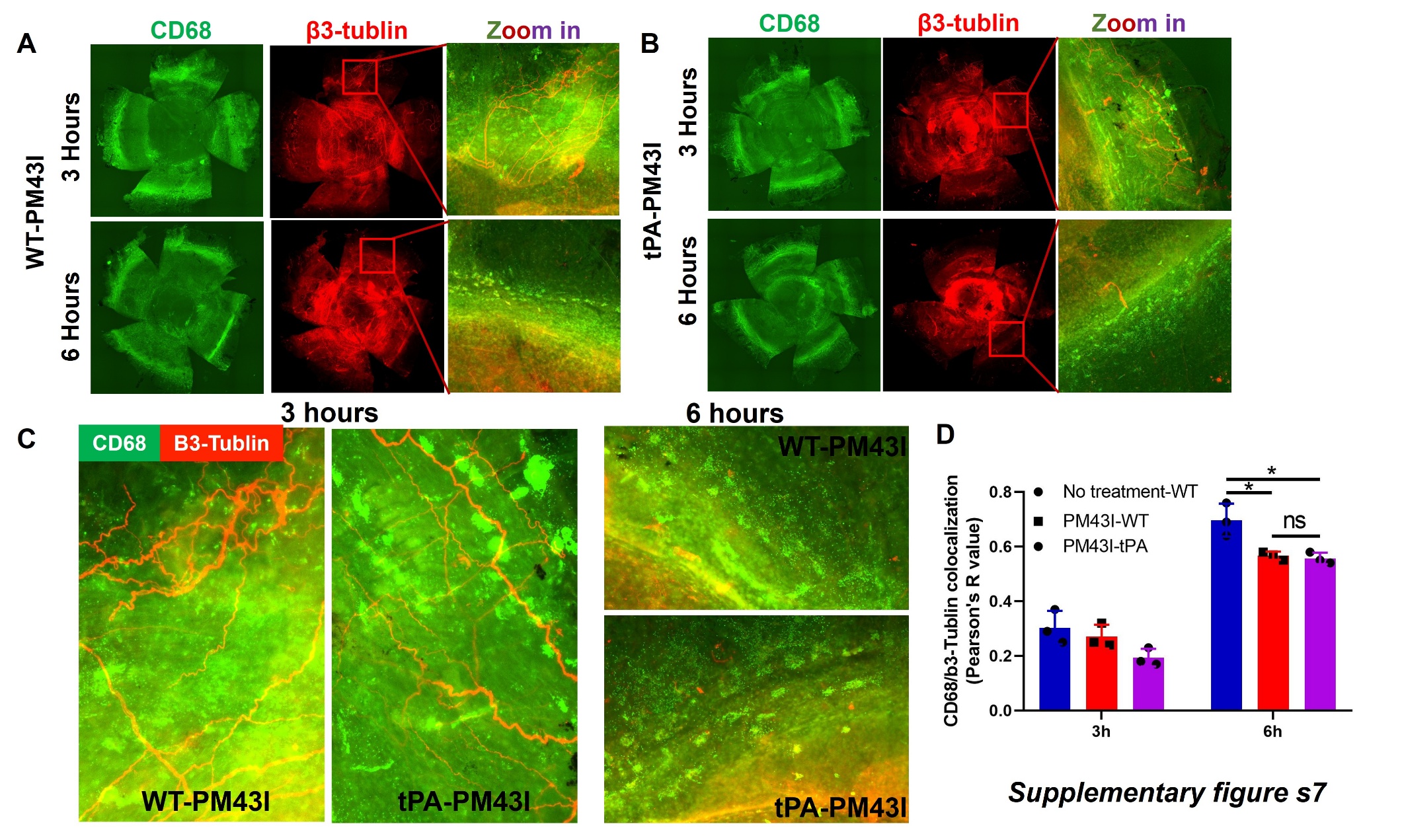


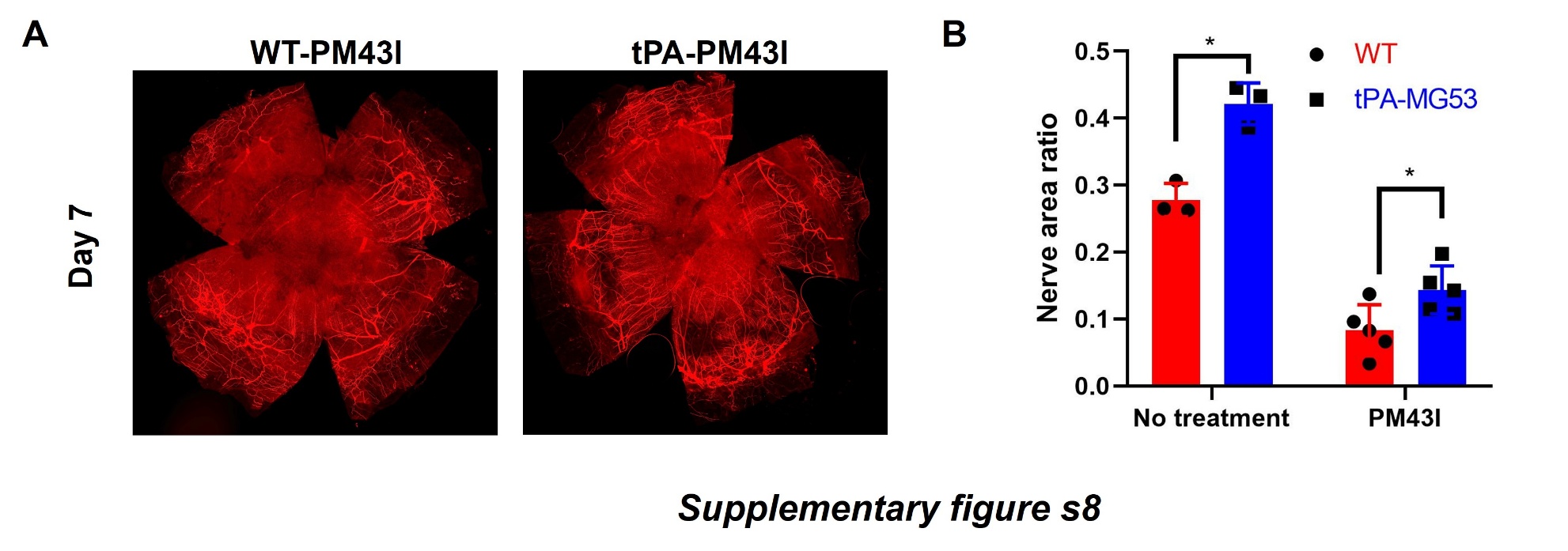


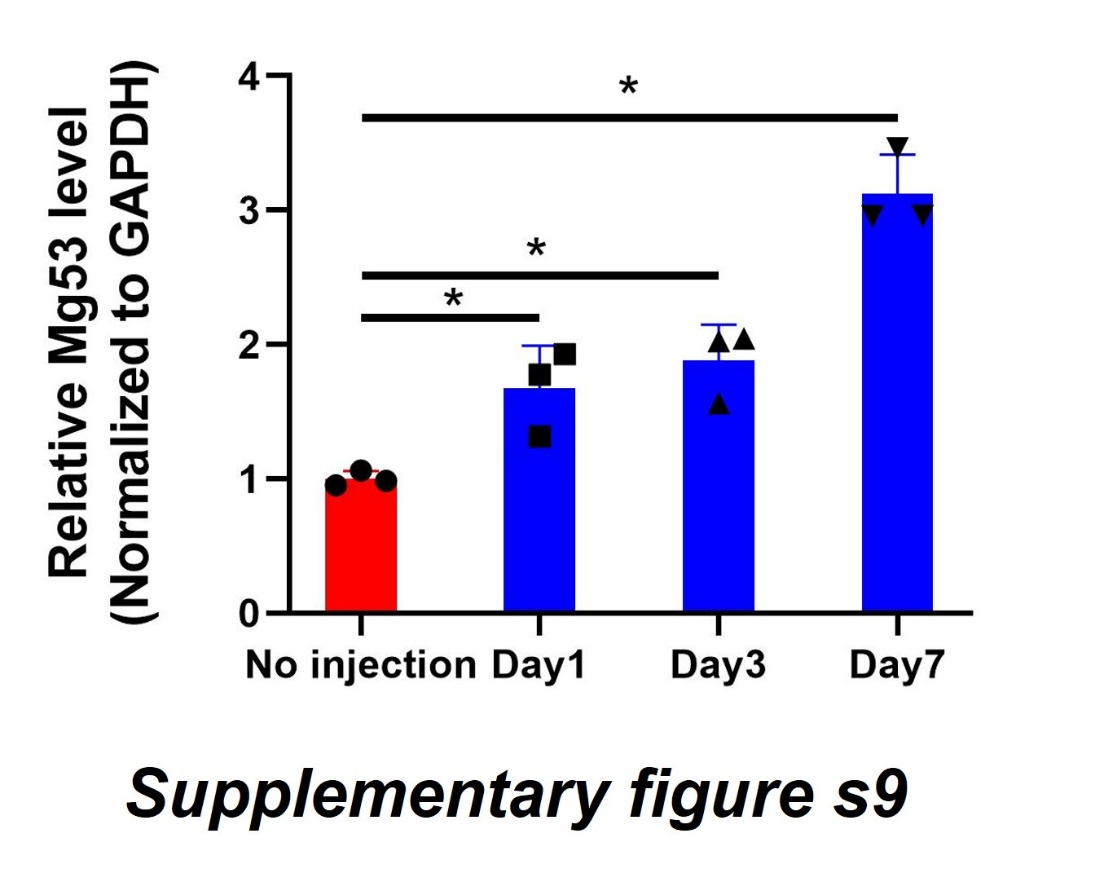


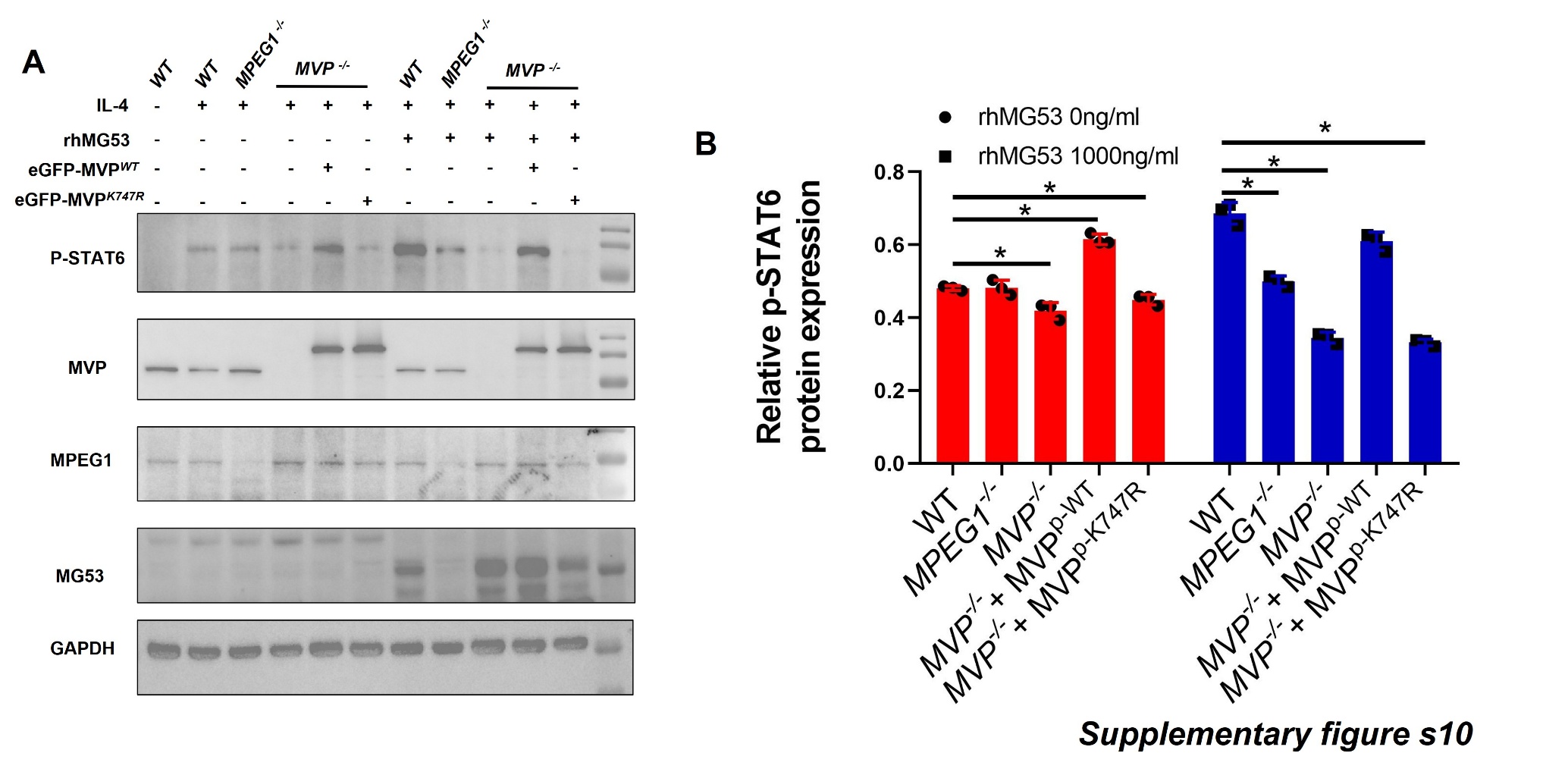

Supplement: Supplementary file 1 — Supporting File 1: advs75206‐sup‐0001‐SuppMat.docx. [file ADVS-9999-e23002-s003.docx]
